# Psychometric validation of the Young Parenting Inventory - Revised (YPI-R2): Replication and Extension of a commonly used parenting scale in Schema Therapy (ST) research and practice

**DOI:** 10.1371/journal.pone.0205605

**Published:** 2018-11-07

**Authors:** John Philip Louis, Alex M. Wood, George Lockwood

**Affiliations:** 1 Stirling Management School, University of Stirling, Stirling, United Kingdom; 2 Department of Psychological and Behavioural Science, London School of Economics and Political Science, London, United Kingdom; 3 Schema Therapy Institute Midwest, Kalamazoo, Michigan, United States of America; Australian National University, AUSTRALIA

## Abstract

This study aimed at developing a revised validated version of the Young Parenting Inventory (YPI) known as YPI-R2 that had 17 theoretical subscales. Using separate ratings for fathers and mothers samples from Singapore (*n* = 582, 617), Manila (*n* = 520, 538), Jakarta (*n* = 366, 383), and the USA (*n* = 204, 214), exploratory and confirmatory factor analysis (CFA) were conducted. This resulted in five subscales for fathers and six for mothers. The 17 theoretical subscales were not supported. Construct, convergent, and divergent validity of this new revised alternative YPI-R2 were also demonstrated. The stringent incremental validity test showed that the YPI-R2 accounted for additional statistically significant variance over and above that contributed by gender and three other established parenting instruments in predicting clinically relevant outcomes. Partial invariance of its factor structure was demonstrated through multigroup CFA using Eastern and Western samples. Finally, significant correlations with the 18 Early Maladaptive Schemas (EMSs) supported a central tenet of schema therapy that these are associated with early negative parenting patterns. Parenting norms in both Eastern and Western cultures that were associated with ill-being were also discussed thus showing the cross-cultural relevance of the YPI-R2.

## Introduction

Schema Therapy (ST) evolved out of decades of clinical experience with helping patients overcome a broad range of deeply entrenched negative core beliefs known as Early Maladaptive Schemas (EMSs; or known colloquially as negative schemas). It is rapidly evolving and attracting empirical tests, initially from within the clinical psychology community; these EMSs have been found to be associated with a variety of psychopathologies, including personality disorders such as borderline personality disorder (BPD) [1–5]. EMSs are broad, pervasive themes comprising emotions, cognitions, memories, bodily sensations, and distorted beliefs about one’s self and others [6]. The theory underlying ST postulates that EMSs develop when the core emotional needs of a child are not met adequately through specific early negative parenting patterns of the caregivers [7, 6]. This tenet of ST is supported by studies showing that EMSs are linked to early negative parenting experiences [8–13]. The association between EMSs and negative parenting patterns or Exasperation Interactions [14] mirrors the important empirical associations found between Early Adaptive Schemas (EASs; or known colloquially as positive schemas) and positive parenting constructs or what we call Nurturing Interactions from recent studies conducted by Louis, Wood, Lockwood, Ho, and Ferguson [15], and Louis, Wood, and Lockwood [16]. To date, 18 EMSs have been identified [17]; their hypothesized relationships with early negative parenting patterns and core emotional needs are shown in S1 Table [7].

The degree and pervasiveness of these unmet needs, in interaction with secondary factors such as quality of the parents’ marriage, culture, and a child’s own temperament [14, 6], determine the severity and strength of these EMSs. For example, a child whose need for warmth, affection and understanding (a specific need within the Connection and Acceptance category) is not adequately met through a nurturing caregiver, is likely to develop an EMS labelled Emotional Deprivation. This child would likely be more prone to experience sadness, depression, anxiety, and/or anger and to cope with this deprivation and associated emotional pain by passively submitting to the mistreatment, fighting back against it, numbing or disconnecting from people and the painful feelings, or a combination of all of these responses. These three main types of coping strategies end up perpetuating EMSs. Usually several EMSs are involved in clinical disorders and, in the case of BPD, almost all of them. ST’s core theory is that these disorders can be successfully treated through, among other things, identifying the associated EMSs, as well as understanding the early negative parenting patterns. These early patterns, which had thwarted their core emotional needs from being met, can now be explored and corrected within the therapeutic relationship, and eventually, with the significant people in their lives [2, 6].

Since recollections of early negative childhood experiences are central to the healing process in ST [6], it is essential for clinicians to have a validated instrument measuring early patterns of parenting that revolve around core emotional needs (S1 Table). To address this issue, Young et al. [6] developed the Young Parenting Inventory (YPI). The development of this measure was based on the hypothesis that each EMS measured by the Young Schema Questionnaire (YSQ [18]) corresponds to a negative pattern of parenting (measured by a subscale in the YPI; see S1 Table) that led to a specific core emotional need not being met. Therefore, each EMS measured in the YSQ scale can be mapped one-to-one with its corresponding pattern of negative parenting measured in the YPI scale. To date, 18 EMSs (in the latest version of the YSQ, the YSQ-S3 [17]) have been identified, but the hypothesized negative parenting pattern associated with the EMS of Social Isolation was not included in the YPI by Young et al. [6] due to the belief that Social Isolation EMS was primarily attributable to external environmental factors rather than negative parenting experiences. Therefore, according to Young et al. [6], there are 17 negative parenting patterns, each believed to be associated to the development of a specific EMS in the YSQ-S3. However, the results from Sheffield, Waller, Emanuelli, Murray, and Meyer [19] did not support this one-to-one mapping of the 17 subscales, finding that the factor structure from the YPI consisted of only nine factors. The aim of this replication paper is to test whether the hypothesis of Young et al. [6] of the 17 one-on-one mapping or the nine factor model [20, 19] can be supported, and if not, to develop a new factor structure that will stand up to full psychometric scrutiny in both Eastern and Western cultures. This replication is important given the emerging use of this scale in ST practice and personality research; as predictions from these fields are tested, such tests must be based on psychometrically reliable and valid measurements.

Several other measures for the assessment of past parenting patterns are widely utilized outside ST. The s-EMBU (Swedish acronym for “My memories of upbringing”) [21] is one of the most widely used and has a strong base of empirical support. These patterns have consistently been grouped into three main subscales on the basis of factor analyses of the s-EMBU. The subscales are named Rejection, Emotional Warmth, and Overprotection. Similarly, the adult version of the Parental Acceptance-Rejection Questionnaire (PARQ) [22] has four subscales; the Parental Authority Questionnaire [23] has three subscales; and the Childhood Trauma Questionnaire (CTQ) [24] has five subscales. While these broad parenting constructs have proven to be extremely valuable, it is possible that, based on the distinctions that form the basis of clinical work in ST, parenting constructs can be more precisely delineated. For example, a construct referring to “rejection” is commonly found in these established subscales. However, rejection from the vantage point of the framework of parenting patterns that failed to meet the core emotional needs, as outlined in S1 Table, could be linked to several different parenting patterns. Thus, a child may feel rejected due to a parent not supporting age-appropriate autonomy, criticizing the child for not living up to academic standards, punishing a child whenever s/he made a mistake, or being absent and inattentive. If these kinds of distinctions prove to have an empirical basis, this will be an important step towards identifying more specific forms of negative parenting patterns which, in turn, will provide a better base for exploring the links between specific parenting patterns and EMSs. It is also likely to lead to an increase in therapeutic leverage and provide a more effective guide for training parents about how they may inadvertently convey a broader theme such as rejection to children.

### Overview of the YPI

The YPI was developed to assess parenting patterns that are hypothesized to lead to the development of EMSs. Rather than the three to five subscales from other established parenting instruments, it hypothesized 17 such subscales, each linked to an EMS measured by the YSQ (see S1 Table). Even if half of these hypothesized subscales can form a reliable factor structure, it would still contain more negative parenting constructs than are found in these other established parenting instruments. This would suggest that the clinical base from which the YPI item pool is derived is providing a more nuanced and potentially broader window into the universe of early toxic parenting patterns, and that by using EMSs, ST can potentially provide a clear vantage point to explore them.

While the YPI has the potential to reveal more negative parenting patterns than other established instruments, only preliminary validation of this instrument was demonstrated by Sheffield et al. [19]. Although this investigation was a significant step forward, it had several important limitations. First, the critical decision of how many factors to extract from the YPI items was based on those with eigenvalues >1.0 rather than Parallel Analysis (PA), which has been shown to more correctly and robustly identify factor structure [25]. Second, the factor structure was never replicated on another independent sample, or tested through Confirmatory Factor Analysis (CFA). Third, the ability of factor analyses to detect valid and reliable factors depends on the initial item pool having enough good quality items to allow a potential factor to emerge [26]. Unlike the related YSQ, which began with 205 items [27, 28] and was then shortened as the scale was refined into the latest version (YSQ-S3) comprising 90 items, the YPI began and ended with the same number of items and never went through a process of scale refinement. Given these reasons there is high risk that the factor structure will not replicate, nor will the evidence of reliability and validity. The only other study that investigated the factor structure of the YPI was a European study that found seven subscales (This study was not translated into English except for the abstract [29]). This is a danger to the emerging research area, as this scale is being used, and research is being conducted globally, with the assumption that all 17 YPI subscales have been validated (e.g. India [30], Iran [31], Palestine [32]). Furthermore, a study in Turkey assumed 10 factors [33] without explanation, and a study in Brazil removed 23 items [34] without any empirical support. Such ongoing research raises further concern about whether the properties of the YPI will replicate across cultures.

One probable reason why the factor structure of the YPI has been assumed to be 17 is due to the theoretical assumption of ST of the one-to-one correspondence between the subscales making up the YPI and the YSQ-S3 subscales measuring the 17 EMSs, because each EMS is assumed to emerge from a negative parenting style. This assumption possibly demotivated a more thorough development of the YPI and, as a result, the factor structure upon which the YPI should be based was never properly developed and established. Further, the early negative parenting pattern associated with the EMS of Social Isolation/Alienation was not included in the YPI subscales because it was not believed to be a result of early interactions with parents but, rather, of later outside-family experiences during adolescence [6]. This was, however, something that should have been shown empirically rather than just assumed.

### The present research

This paper comprises three phases that attempt to replicate Young et al.’s [6] hypothesized 17-factor model, as well as Sheffield et al. [19] nine-factor model, labeled as YPI-R, and in finding them to be inadequate, revises the YPI from the item development stage onwards in line with established psychometric principles [35]. In Phase 1 the aim was to investigate the factor structure of the YPI, using PA in determining the number of factors to be retained. A reliable factor structure was identified, but one that neither replicated Sheffield et al. [19] nor conformed to the theoretical model of Young et al. [6]. The factor structure consisted of both strong and weak subscales, with the latter defined by lower-loading items of two or less. To determine whether the failure to replicate emerged from a small item pool, new items were developed by an experienced team. Phase 2 developed a new, shorter revised scale of the YPI, known as YPI-R2, which represents the core EMS-related parenting styles. In Phase 3 this new factor structure was established and tested on both an Eastern and Western sample. The scale also demonstrated convergent, divergent, construct validity and incremental validity above other parenting scales in predicting clinically relevant outcomes. For evidence of construct validity, established parenting subscales were compared with those of YPI-R2. Positive correlations of moderate strength (*r* = .3 to .6) were expected between subscales from these established measures of negative parenting patterns with subscales of the YPI-R2 that shared similar constructs. For example, subscales that measure various facets of Rejection would have the highest positive correlations with a subscale of the YPI-R2 that most represents this construct. Likewise, the positive construct of Warmth from other established parenting scales was expected to correlate the highest but negatively with the most nonconcordant construct of Warmth in the YPI-R2. For convergent validity, since studies have shown that the quality of relationship between parents and child shape their personality development, and is linked to emotional distress and psychological well-being over time, we expected positive correlations of moderate strength between subscales of YPI-R2 with negative personality dispositions and emotional distress [36, 37, 22, 4]. Conversely, we expected negative correlations of the same strength with the positive measures gratitude and psychological well-being [38, 39].

Divergent validity was tested based on the a priori assumption that the subscales of the YPI-R2 that were less concordant with subscales of other established parenting measures would be less strongly correlated (since they are capturing a less common construct) than those that were more so. The YPI-R2 was also subjected to a test of incremental validity in order to show that this newly developed scale was not yet another addition to the proliferation of negative parenting scales that measure the same constructs, but that it would contribute uniquely and separately to the prediction of psychological well-being, emotional distress, personality disposition, and positive traits, above and beyond what can be predicted by these other established parenting scales. Finally, this scale with negative parenting constructs also showed convergent validity through statistically significant associations with EMSs, lending strong support for the tenet of ST that negative parenting patterns are associated with the development of EMSs. This was similar to associations found between EASs and positive parenting constructs [15, 16]. Out of failure to support the expected 17- and nine-factor structure, a unique new scale emerges with broad applicability to many forms of psychotherapy and lines of research and with special relevance to those involved in ST research and practice.

## Method

### Samples

Nonclinical community samples made up of English speaking singles, students, and parents were drawn from a pool of volunteers from nongovernmental organizations (NGOs) located in three Southeast Asian cities (Eastern samples); Singapore, Manila (Philippines), Jakarta (Indonesia), as well as from three cities in the East coast of the United States (Western sample); Fairfax and Stafford located in Northern Virginia, and Manchester located in New Hampshire. These NGOs were part of an international charity headquartered in the USA, and approval was obtained by the ethics committee of each NGO, and by the [blinded] Management School ethics committee. Ethical considerations were in accordance with the British Psychological Society. The purpose of the research, the voluntary nature of their involvement and other information were sent to all participants via email, by distribution of hard copies, as well as online invitations through advertisements in their websites. Invitations to take part were also sent to other organizations in these cities, whereby volunteers were encouraged to reach out to friends. As a result, samples were drawn from populations consisting of professionals, students, and parents. Workshops on the effects of past parenting behavior and the development of schemas were conducted without charge as incentive for all participants. No volunteers from this NGO in any city were excluded because of race, color or religion. Participants signed on the first page of the questionnaire to grant consent. The only types of participants that were excluded were those below 18 years of age and those who did not have an adequate command of the English language. Sufficient grasp of the English language was determined by both polling members of the respective groups and the lead researcher’s familiarity with the leaders of these respective groups and their familiarity with the members of the respective NGOs. The mean age of the Singapore sample was 36.99 years (*SD* = 7.87); of the Manila sample, 41.60 years (*SD* = 11.90); the Jakarta sample, 37.10 years (*SD* = 11.80); and the USA sample, 43.40 years (*SD* = 22.60). Analyses for fathers and mothers were conducted separately, for which the values of *n* were as follows: Singapore ratings of fathers (*n* = 582) and mothers (*n* = 617); Manila ratings of fathers (*n* = 520) and mothers (*n* = 538); Jakarta ratings of fathers (*n* = 366) and mothers (*n* = 383) and; USA ratings of fathers (*n* = 204) and mothers (*n* = 214). The demographic characteristics of these samples are presented in S2 Table.

### Instruments

#### YPI

This scale has 17 theoretical subscales (72 items) that retrospectively measures perceived parenting experiences of an adult using a 6-point Likert scale, ranging from 1 (*Completely untrue*) to 6 (*Describes him/her perfectly*) (see S1 Table for item examples). Scores on each sub-scale of the YPI are provided separately for ratings of fathers and mothers, or those whom the participants considered as having assumed a paternal or maternal role (grandparent, step mother or father, or a much older sibling), as different patterns of correlations may emerge depending on the gender of the parent who is adopting a particular parenting style. Young hypothesized a 17-factor model where each subscale in the YPI was associated with the development of one specific EMS measured by the YSQ-S3. However, another factor structure emerged from Sheffield et al. [19] with nine subscales; Emotionally Depriving, Overprotective, Belittling, Perfectionist, Pessimistic/Fearful, Controlling, Emotionally Inhibited, Punitive and Conditional/Narcissistic. Cronbach’s alpha reliability values ranged from .67 to .92. Acceptable test-retest reliability and correlations ranged from .53 to .85. Construct validity was shown with the YSQ measuring 15 EMSs. The goodness of fit of Young’s 17-factor model as well as nine-factor model was also investigated.

#### s-EMBU (a Swedish acronym for “My memories of upbringing”) short version

This is a 23-item self-report inventory designed to measure adults’ perceived parenting experiences [21]. There are three subscales: Rejection, “It happened that my parents gave me more corporal punishment than I deserved”; Warmth: “My parents praised me”; and, (Over) Protection: “It happened that I wished my parents would worry less about what I was doing”. Items are answered on a four-point scale with reference to father and mother separately, ranging from 1 (*No*, *never*) to 4 (*Yes*, *most of the time*). The reliability values were from a study conducted across four European countries where, *α* = .72 to .85, for fathers and mothers [36]. The correlation of at least one subscale of s-EMBU with scales of the Eysenck Personality Questionnaire Revised-Abbreviated [40], and Rosenberg Self-Esteem [41] was above, *r* = .30. It was expected that the YPI-R2 (Fathers) and YPI-R2 (Mothers) would demonstrate construct validity with similar subscales of s-EMBU with moderate size correlations.

#### Parental Acceptance-Rejection Questionnaire (PARQ) adult version

This instrument assesses adults’ perceptions of their mothers’ and fathers’ behavior towards them when they were growing up [22]. Each of the 24 items in this instrument has a four-point Likert scale, from 1 (*Almost always true*) to 4 (*Almost never true*). Item examples: Warmth / Affection: “Made me feel wanted and needed”; Hostility / Aggression: “Went out of her way to hurt my feelings”; Indifference / Neglect: “Paid no attention to me as long as I did nothing to bother her”; Undifferentiated / Rejection: “Did not really love me”. The reliability coefficients were reported to be at least from, *α* = .86 to .95 and convergent, divergent and construct validity was shown [22] in correlations with three subscales from Children’s report of Parent Behavior Inventory [42] measuring acceptance, hostility and rejection, (*r* ≥ .81), and one sub-scale of Bronfenbrenner’s Parental Behavior Questionnaire [43] measuring punishment (*r* = .43). This instrument has often been used in conjunction with the PAQ to investigate links between parenting and personality dispositions. The mean effect sizes of statistically significant correlations of maternal and paternal acceptance and with at least one of the PAQ sub-scale was, *r* = .39. It was expected that the YPI-R2 (Fathers) and YPI-R2 (Mothers) would demonstrate construct validity with similar subscales of PARQ with moderate strength correlations.

#### The Childhood Trauma Questionnaire (CTQ)

This instrument contains 28 items that measures past parenting experiences on five types of childhood abuse or neglect; Emotional Abuse: “I thought my parents wished I had never been born”); Physical Abuse: “I got hit so hard by someone in my family that I had to see a doctor or go to the hospital”); Sexual Abuse (“Someone molested me”); Emotional Neglect (reverse score; e.g., “I felt loved”); and, Physical Neglect (“My parents were too drunk to or high to take care of the family”). This is also an optional subscale called Minimization / Denial (“I had the perfect childhood). Items are answered on a five-point Likert scale, ranging from 1 (*Never true*) to 5 (*Very often true*). The reliability values for the CTQ subscales ranged from, *α* = .79 to .94. It also demonstrated good test-retest reliability over a 2- to 6-month interval (intraclass correlation, *r* = .88). The psychometric validation of this instrument has been shown in many samples [24, 44]. The parenting subscales of YPI-R2 (Fathers) and YPI-R2 (Mothers) were expected to show construct validity with parenting subscales of the CTQ with moderate size correlations.

#### Adult version of Personality Assessment Questionnaire (PAQ)

This scale consists of seven personality dispositions with 63 items and each has a four-point Likert scale, from 1 (*Almost always true*) to 4 (*Almost never true*) that assesses individuals’ perceptions of themselves with respect to seven personality dispositions). Items examples: Hostility / Aggression: “I have trouble controlling my temper”; Dependency: “I like to be given encouragement when I have failed”; Negative Self-Esteem: “I wish I could have more respect for myself”; Negative Self-Adequacy: “I feel inept in many of the things I try to do”; Emotional Unresponsiveness: “I feel distant and detached from most people”; Emotional Instability: “I am cross and grumpy without any good reason”; Negative Worldview: “I view the universe as a threatening, dangerous place”. Collectively the seven PAQ subscales represent a measure of respondents’ overall psychological adjustment, which was predicted to be associated with the experience of parental acceptance-rejection [22]. This instrument has been psychometrically validated [45] in Asian and Western samples. The Alpha coefficient of the Adult PAQ was reported to be at least, *α* ≥ .80 [46]. Convergent validity was shown with other scales similar in constructs such as Buss and Durkee’s Hostility [47], and Shostrom’s Self-Regard [48]. The correlation values ranged from, *r* = -.50 to -.83 [22]. For convergant validity, it was expected for the correlations between subscales of YPI-R2 (Fathers) and YPI-R2 (Mothers) to be of moderate size (*r* = |.20| to |.40|), as previous research using parenting scales has shown correlations of these sizes with personality and self-esteem [36].

#### Ryff’s scales of psychological well-being

This version consists of six subscales and 18 items (three items per scale), each answered on a five-point Likert scale ranging from 1 (*Strongly disagree*) to 5 (*Strongly agree*). This instrument measures six facets of psychological well-being; Positive Relations with Others: “Maintaining close relationships has been difficult and frustrating for me”; Autonomy: “I tend to be influenced by people with strong opinions”; Personal Growth: “I think it is important to have new experiences that challenge how you think about yourself and the world”, Environment Mastery: “I am quite good at managing the many responsibilities of my daily life”; Purpose in Life: “Some people wander aimlessly through life, but I am not one of them”; Self-Acceptance: “I like most aspects of my personality” [49]. These six subscales were regarded as measures of psychological well-being form a latent “psychological well-being” variable, which is highly correlated (*r* > .20) with but distinct from “subjective well-being”, consisting of positive and negative affect, and life satisfaction [50]. In this study the reliability values for the six subscales ranged from, *α* = .69 to .81. For convergent validity, it was expected that the correlations with subscales of PAQ to be of moderate size (*r* = |.20| to |.40|), as previous research using parenting scales has shown correlations of these sizes with personality and self-esteem [36].

#### The depression, anxiety, stress scales (DASS-21)

The DASS-21 contains 21 items measured on a four-point Likert scale, ranging from 0 (*Did not apply to me at all*) to 4 (*Applied to me very much or most of the time*). There were three subscales: Depression: “I couldn’t seem to experience any positive feeling at all”; Anxiety, “I experienced trembling (e.g. in the hands)”; and Stress: “I found it hard to wind down”. The instrument was validated by Antony, Bieling, Cox, Enns, and Swinson [51]. Cronbach’s alpha reliability values for depression, anxiety and stress were .94, .87 and .91 respectively. Correlations values with other measures of depression, anxiety and stress such as Beck Depression Inventory [52] where, *r* = .62 to .79); Beck Anxiety Inventory [52] were, *r* = .51 to .85. It was expected that correlations between YPI-R2 (Fathers) and YPI-R2 (Mothers) with DASS-21 to be of moderate size (*r* = |.20| to |.40|) for convergent validity.

#### The Gratitude Questionnaire (GQ-6)

The GQ-6 [53] is a six-item questionnaire that measures the disposition to experience gratitude. Each item is answered on a seven-point Likert scale ranging from 1 (*Strongly disagree*) to 7 (*Strongly agree*). Item examples, “Long amounts of time can go by before I feel grateful to something or someone”; “As I get older I find myself more able to appreciate the people, events, and situations that have been part of my life history”. The GQ-6 scale [54] showed substantial incremental validity in predicting both life satisfaction and psychological well-being [55] above the 50 facets of the Five Factor Model. It also longitudinally predicts decreases in stress (*r* = -.30 to .42) and depression (*r* = -.48), and increases in social support (*r* = .29 to .60), over three months, during which the GQ-6 exhibited a test-retest reliability between, *r* = .58 and .73 [56]. It was expected that correlations between YPI-R2 (Fathers) and YPI-R2 (Mothers) with GQ-6 to be of moderate size (*r* = |.20| to |.40|) as demonstration of convergent validity.

#### Young Schema Questionnaire (YSQ-S3)

The latest measure of EMSs, the YSQ-S3, measures 18 EMSs, and has a 6-point Likert scale that ranges from a score of 1 (*Completely untrue of me*) to a score of 6 (*Describes me perfectly*). Item examples: For Mistrust / Abuse negative schema, “I feel that people will take advantage of me”; Defectiveness / Shame negative schema, “No man/woman I desire could love me once he/she saw my defects”. The YSQ-S3 was validated [57] using community as well as clinical samples. Internal consistency were >.70 for 17 of the EMSs, except for the Entitlement EMS (.67). Cronbach’s alpha reliability values for 17 EMSs were satisfactory (>.70) except for Entitlement. Another study [58] also validated the YSQ-S3, where all 18 EMSs had a Cronbach’s alpha reliability value of >.70. The subscales of the YSQ-S3 were also meaningfully associated with personality disorders. It was expected that the convergent validity of the final YSQ-S3 subscales measuring EMSs would be demonstrated through positive correlations with YPI-R2 (Fathers) and YPI-R2 (Mothers), with positive correlations ranging from, *r* = |.20| to |.40| since similar results emerged between EMSs and a parenting scale in a study by Thimm [4].

## Procedure and statistical analyses

IBM SPSS Statistics 23 [59] was used to conduct Exploratory Factor Analysis (EFA), compute Pearson’s correlations and Cronbach’s alpha reliability values, and run hierarchical regression analyses. For single and multigroup CFA, M*plus* 8 software using Weighted Least-Squares Mean and Variance (WLSMV) adjusted estimations was used [60] since we modeled these data to account for the ordered-categorical nature of the response scales [61]. A missing data analysis was initially carried out using Little's Missing Completely at Random test (MCAR) [62] to see if missing patterns were at random. A robustness check was carried out on the analysis based on ratings of the fathers to determine the impact of missing values on the data. Three methods were employed to investigate this–Exclude case pairwise feature in SPSS, replacing missing data with the mean value, and Multiple Imputation, using the 5^th^ imputed data set. If no differences emerged from the factor structure from all three methods, then the mean of all responses from other subjects was used to impute the missing values.

Initially, a CFA was conducted to test the goodness of fit of the 17-factor model of Young’s hypothesis [6], as well as the nine-factor model from Sheffield et al. [19]. If these factor structures could not be replicated in this sample, an EFA using Principal axis factoring (PAF) was to be conducted to investigate its factor structure. The suitability of the sample data for EFA was determined using the Kaiser-Meyer-Olkin (KMO) test and Bartlett’s test of Sphericity. The number of factors to be extracted from the data was determined using PA, because this method is more accurate at detecting the true number of factors in data than other commonly used methods [25]. Based on a recommendation by Tabachnick and Fidell [63, 64], we determined to use an oblique method (promax) rather than an orthogonal rotation if values of the factor correlations matrix were .32 and above. Factor correlations were also inspected to see if there was an overlap between factors. The item selection criteria used to select the most robust items to form the shorter form of YPI-R2 were as follows: Items with factor loadings < .40 were dropped [65, 66]. Items that had the highest loading were given priority [36]. Based on recommendation by Floyd and Widaman [65], three to eight items per subscale were selected in order to make it easier for factor structures to be confirmed with CFA. From the EFA results in Phase 2, items with high item-to-item correlations were also removed to ensure that fit indices values were not compromised in subsequent CFA in Phase 3. For Cronbach’s alpha reliability values, according to Clark and Watson [26], only subscales with values above .60 can be viewed as adequate.

These analyses followed the guidelines in which a close fit is indicated by normed chi-square, (*X*^*2*^/*df*) < 4 [67, 68]; the root mean square error of approximation (RMSEA), where a reasonable fit by 0.06 <RMSEA <0.08, a mediocre fit by 0.08 <RMSEA <0.10, and an unacceptable fit by RMSEA >0.10 [69]; comparative fit index (CFI), and one non-normed fit index known as the Tucker-Lewis (TLI) by values ≥ .95 for a good fit and ≥ .90 for an adequate fit [70]. Each model under examination needed to be further evaluated for acceptable fit based on prior findings. Floyd and Widaman [65] found that scales with high numbers of items and factors generally lead to a poorer fit. This was evident from three studies; Bach et al. [58], Baranoff, Oei, Cho, and Kwon [71], and Kriston et al. [57], where the YSQ-S3 (90 items) were subjected to CFA, in which the CFI obtained was below the .9 threshold with values of .84, .87, and .85, respectively (the values of *X*^*2*^/*df* and RMSEA in these studies were above the recommended minimum threshold). Thus more relaxed values for indices may be considered an acceptable fit for such scales; for example, a value for CFI and TLI that is slightly less than .90 can be viewed as a moderate fit in studies with a large number of items. Equally, for scales with a small number of items, it would be appropriate to adopt more stringent fit criteria [65]. Given the number of factors and items, we determined a priori to accept the lower bound of fit values as well fitting in the context. For multigroup CFA (MGCFA) the following measurements of invariance [72] were used for the two independent samples (Jakarta and USA): (1) configural invariance (same factor structure across groups); (2) metric invariance (same factor loadings across groups); (3) scalar invariance (same item intercepts across groups); (4) error invariance (same error variance across groups); (5) factor variance invariance (same factor variance across groups); (6) factor covariance (same factor covariance across groups), and (7) factor mean invariance (same factor mean across groups). The above seven models address full measurement invariance because each of the above components should be equal in both independent samples (Jakarta and USA). Byrne, Shavelson, and Muthén [73] introduced the concept of partial invariance, and for this to be achieved, according to Vandenberg and Lance [74], at least configural and metric invariance need to be established.

Construct and convergent validity were assessed on the Manila sample in Phase 3 using Pearson’s correlations. We adopted conventional guidelines as to what is considered a small (*r* = .10), medium (*r* = .30), and large effect size (*r* = .50 [75]). Rules of thumb were developed for conventional effect sizes for zero-order correlations on the assumption that the relationships would be confounded at least somewhat by third variables; hence effect sizes had to be of a certain magnitude to be considered meaningful. To test divergent validity, we chose the s-EMBU scale as comparison, because it has three varied constructs (Rejection, Warmth and Overprotection) as opposed to the CTQ with only two broad constructs (Emotional and Physical Neglect, and Abuse) each being somewhat concordant, or the PARQ, again, with only two broad constructs (Acceptance and Rejection). The z-test proposed by Steiger [76] was used to show, as evidence for divergent validity, that differences in correlations between most concordant subscales in the YPI-R2 and s-EMBU were statistically and significantly higher than differences in correlations with less concordant subscales of both measures.

Finally, incremental validity was determined using hierarchical multiple regression with guidelines from Hunsley and Meyer [77] who emphasized that rules of thumb (in this case for effect sizes) must be used relative to the context. With good tests of incremental validity, much of the third variable’s effect has been removed. Hence, a minimum of 2.25% (equivalent to *r* = .15) should be considered a “reasonable contribution” [77; pp. 451] and must be achieved from the second to third step of a regression analysis. One of the conditions for regression analysis is that the distribution of data of the dependent variables has to be normal, although both CFA and EFA appear to be robust against violations of this requirement [65] if sample size is ≥ 200 [64], which was the case in this study. The normality of the distribution was confirmed by inspecting values of kurtosis and skewness. According to Hair, Black, Babin, and Anderson [78], and Byrne [79], data for the dependent variables can be considered to be normal if skewness is between -2 to +2 and kurtosis is between -7 to +7.

## Results

### Missing data

For the Singapore, Manila, Jakarta and USA samples, the percentage of missing data was very low: for ratings of fathers, Singapore = .012%, Manila = .63%, Jakarta = .85%, USA = .10%; ratings of mothers, Singapore = .02%, Manila = .67%, Jakarta = 3.27%, USA = .09%. Results from a MCAR test for ratings of fathers: Singapore, Chi-Square = 193.37, DF = 284, *p* = 1.00; Manila, Chi-Square = 86423.57, DF = 84668, *p* = .00; Jakarta, Chi Square = 55811.28, DF = 60342, *p* = 1.00; USA, Chi-square = 2862.74, DF = 2911, *p* = .74. For ratings of mothers: Singapore, Chi-Square = 664.18, DF = 639, *p* = .24, Manila, Chi-Square = 99601.58, DF = 97712, *p* = .00; Jakarta, Chi Square = 66412.72, DF = 68973, *p* = 1.00; USA, Chi-square = 2500.18, DF = 2619, *p* = .95. These patterns of missing data were random except for the Manila sample. However, no variables had an unusually high number of missing values in comparison to the rest. All three methods for imputing missing data (see Section 5.4, Procedures and Statistical Analyses) yielded almost identical EFA results using the Manila ratings of fathers sample, with the same 14 factors (as suggested by PA) and almost the same items under each factor, showing that the impact of missing data was negligible. As a result, the average value of all responses from other subjects was chosen to impute the missing values in all the samples.

### Phase 1 confirmatory factor analysis and exploratory factor analysis of the YPI

A CFA was conducted to test the goodness of fit of the 17-factor model of Young’s hypothesis [6], as well as the nine-factor model from Sheffield et al. [19] on the Singapore sample. For Fathers, *χ*^*2*^ = 14993.9, *df* = 2348, *p* < .001, *χ*^*2*^*/df* = 6.386, RMSEA = 0.096, CFI = 0.668, TLI = 0.639; For Mothers, *χ*^*2*^ = 13028.2, *df* = 2348, *p* < .001, *χ*^*2*^*/df* = 5.549, RMSEA = 0.086, CFI = 0.731, TLI = 0.707). For the Sheffield et al. [19] nine-factor model, the CFA indices were: for Fathers, *χ*^*2*^ = 5645.53, *df* = 593, *p* < .001, *χ*^*2*^*/df* = 9.520, RMSEA = 0.121, CFI = 0.697, TLI = 0.660; For Mothers, *χ*^*2*^ = 4695.51, *df* = 593, *p* < .001, *χ*^*2*^*/df* = 7.918, RMSEA = 0.106, CFI = 0.768, TLI = 0.739. Since neither factor structures could be replicated, an EFA was conducted on this Singapore sample. For the ratings of the fathers, the KMO index was .94, and Bartlett’s test of Sphericity was statistically significant, χ^2^ (2556, *n* = 582) = 22500.69, *p* < .001, showing that two basic assumptions of factor analysis were met. Similarly, for the ratings of the mothers, the KMO index was .94, and Bartlett’s test of Sphericity was statistically significant, *χ*^2^ (2556, *n* = 617) = 23710.89, *p* < .001, again showing the suitability of factor analysis. PAF with oblique (promax) rotation was used, since many values in the factor correlation matrix were greater than .32 [63]. PA recommended 13 factors to be extracted from both the father and mother samples. For the fathers, this accounted for 52.29% of total variance. The 10^th^ factor had two items but one of them cross loaded heavily (>.30) with another more robust factor; the 11^th^ factor had only one item; the 12^th^ factor had two items that cross loaded heavily with another more robust factor; the 13^th^ factor had no items that loaded more than .40. Thus these four factors were rejected, leaving only nine factors in the ratings of the fathers that could be considered for further analysis. For the mother sample, 13 factors accounted for 51.64% of the total variance. The 11^th^ factor had two items, both of which shared very similar constructs with a more robust factor; the 12^th^ factor had only one item; the13^th^ factor had no items at all with factor loadings more than .40. As a result these three factors were rejected, and only 10 factors were considered for further analysis. The average factor correlations were .23 and .26 for ratings of fathers and mothers, respectively.

Based on the item selection criteria (see Procedures and Statistical Analyses), six factors were considered weak because their Cronbach’s Alpha values were below .60 [26] and/or because they had fewer than three items with loadings > .40 [65]. These were labeled Pessimism (father and mother), Undependability and Irresponsibility (mother), Fear of Harm and Illness (father and mother), Overindulgence (mother), Unstable (father), and Dependent and Worrisome (mother). Four robust subscales were common to both the ratings of the fathers and those of the mothers: Competitiveness and Status Seeking, Emotional Inhibition and Deprivation, Degradation and Rejection, and Overprotection. Two additional robust subscales from just the ratings of the fathers were Undependability and Irresponsibility, and Overindulgence; and one additional scale, labeled Punitiveness, was unique to ratings of the mothers. These robust subscales had reliability values that ranged from .70 to .92.

Thus in Phase 1, the factor structure of Young’s 17-factor model [6], as well as the nine-factor model from Sheffield et al. [19], could not be replicated on the Singapore sample. This justified conducting an EFA of the YPI, yet results did not yield a robust factor structure, as there were six weak factors. Therefore, Phase 2 had two aims. The first was to expand the YPI item pool with new items to strengthen the weaker subscales from Phase 1, augment the stronger subscales, and measure the one missing subscale (Social Isolation). The second was to refine this initial item pool through factor analytical work, followed by an item selection process (see Procedures and Statistical Analyses) of the most robust items for each subscale [36, 65], as emerging scales should contain only the most representative items.

### Phase 2 initial item pool development

To develop a larger initial item pool of the YPI, a competent team of four individuals was formed, each an expert in his field. The first three of the four are members of the International Society for Schema Therapy (ISST). Two had held board positions in the ISST, whilst the fourth was fully independent and prior to this project had no knowledge of ST or the underlying theory (although he is an expert in other therapeutic approaches that were antecedent to ST). The process of development included forming consensus, which took about one month. Through this process, an initial item pool of 204 negative parenting items (72 items from the original YPI, and 132 new items) representing 18 EMSs were formed, including those representing the EMS of Social Isolation. Each item followed the same Likert scale as in the original YPI. Item examples for the construct of Social Isolation are, “Was (seemed to be) jealous of my friends”; “Discouraged me from inviting friends to our house”.

### Phase 2 exploratory factor analysis of initial item pool of the YPI

EFA was performed on the Manila data for the father and mother samples separately. For the ratings of the fathers, the KMO index was .92, and Bartlett’s test of Sphericity was statistically significant, χ^2^ (20706, *n* = 520) = 59483.38, *p* < .001. For the ratings of the mothers, the KMO index was .92, and Bartlett’s test of Sphericity was statistically significant, χ^2^ (20706, *n* = 538) = 59045.18, *p* < .001. Therefore, data from both samples were suitable for factor analysis. Results of PA and EFA of the ratings of fathers using the oblique (promax) rotation produced a 14-factor solution that accounted for 39.46% of the total variability. Out of the 14 factors, five had only 1 or 2 items. One factor had three items, but these items represented very similar constructs as another more robust factor. Therefore, six factors were removed, leaving eight factors for further analysis. The PA and EFA for the ratings of mothers produced a 13-factor solution that accounted for 37.67% of the total variability. Of these, five factors had two or fewer items. These five factors were rejected, leaving eight factors for further analysis. When results for ratings of fathers and mothers were compared, each had eight factors; six were common factors (Degradation and Rejection, Competitiveness and Status Seeking, Emotional Inhibition and Deprivation, Overprotection and Overindulgence, Punitiveness, and Undependability and Irresponsibility). Two additional factors were unique to the fathers (Dependency and Social Isolation, and Intrusiveness and Exploitation), and two to mothers (Fear of Harm and Illness, and Controlling; see S3 Table for EFA results with cut off points of >.4). Before this factor structure could be tested for goodness of fit on the Jakarta sample, the Cronbach’s alpha reliability values for these eight factors were tested on both the Manila and Jakarta samples. All subscales had values > .6 except for two subscales in the Jakarta sample: Intrusiveness and Exploitation for the fathers, and Undependability and Irresponsibility for the mothers, which were .55 and .54, respectively, both below the .6 mark. Both these subscales were therefore rejected, leaving seven subscales for ratings of fathers and mothers. Inter-factor correlations of the YPI-R2 were mostly low to moderate, and the highest in both samples were .60 and .64 for ratings of fathers and mothers (S4 and S5 Tables), respectively, indicating absence of overlap between factors [26] (see Table 1,) or problems associated with multicollinearity. Thus in Phase 2, seven robust factors emerged from the initial item pool of 204 items for ratings of both fathers and mothers; in Phase 3, this factor structure was tested using CFA with an independent sample from Jakarta.

**Table 1:** Phase 3 –Pearson's correlation matrix of the YPI-R2 (Fathers) and YPI-R2 (Mothers) with s-EMBU, CTQ-28, PARQ, PAQ, DASS-21, Ryff's Well-Being, GQ-6 Using Manila Sample (n = 520, 538).

	CSS		DR		EID		OO		PU		CTL	
F: Father / M: Mother	F	M	F	M	F	M	F	M	F	M	F	M
sEMBU—Rejection	.09*	.09*	.53**	.62**	.15**	.28**	.13**	.05	.56**	.62**	-	.51**
sEMBU—Emotional Warmth	.18**	.19**	-.36**	-.46**	-.34**	-.34**	.20**	.15**	-.32**	-.35**	-	-.29**
sEMBU—(Over)Protection	.24**	.26**	.33**	.33**	.10*	.15**	.36**	.27**	.37**	.31**	-	.45**
CTQ-28—Emotional Abuse	.01	.03	.42**	.58**	.16**	.30**	.02	.03	.42**	.54**	-	.43**
CTQ-28—Physical Abuse	.01	.04	.34**	.47**	.08	.24**	.03	.04	.53**	.58**	-	.37**
CTQ-28—Sexual Abuse	-.07	-.04	.13**	.23**	.08	.18**	-.02	-.00	.19**	.25**	-	.20**
CTQ-28—Emotional Neglect	-.09*	-.13**	.35**	.44**	.26**	.33**	-.13**	-.13**	.29**	.34**	-	.26**
CTQ-28—Physical Neglect	-.02	-.11**	.36**	.38**	.11*	.18**	-.07	-.09*	.34**	.32**	-	.30**
PARQ—Hostility/Aggression	.08	.05	.60**	.70**	.22**	.34**	.02	-.03	.68**	.69**	-	.50**
PARQ—Indifference/Neglect	-.07	-.08	.47**	.58**	.37**	.36**	-.18**	-.15**	.46**	.47**	-	.43**
PARQ—Undifferentiated Rejection	.08	-.02	.61**	.69**	.24**	.30**	.02	-.07	.56**	.53**	-	.46**
PARQ—Warmth Affection (Reverse scored)	-.10*	-.17**	.40**	.58**	.42**	.42**	-.20**	-.19**	.40**	.49**	-	.37**
PAQ- Hostility/Aggression	.06	.03	.36**	.38**	.15**	.13**	.05	.06	.28**	.30**	-	.24**
PAQ- Dependency	.04	.02	.03	.01	.01	.00	.08	.03	.14**	.06	-	.03
PAQ- Negative Self-Esteem	-.07	-.12**	.39**	.31**	.10*	.14**	.12**	.10*	.22**	.17**	-	.16**
PAQ- Negative Self-Adequacy	-.08	-.18**	.37**	.26**	.12**	.13**	.18**	.11*	.19**	.15**	-	.11**
PAQ- Emotional Unresponsive	.00	-.06	.27**	.20**	.11*	.20**	.10*	.05	.09	.07	-	.12**
PAQ- Emotional Instability	-.03	-.02	.25**	.22**	.12**	.15**	.10*	.09*	.16**	.15**	-	.15**
PAQ- Negative Worldview	.03	-.06	.37**	.33**	.15**	.15**	.09*	.08	.19**	.15**	-	.19**
DASS-21 –Anxiety	.03	.01	.27**	.21**	-.00	.07	.21**	.14**	.19**	.15**	-	.19**
DASS-21 –Depression	-.01	-.10*	.36**	.25**	.05	.12**	.21**	.16**	.16**	.13**	-	.16**
DASS-21 –Stress	.04	.02	.27**	.26**	.08	.15**	.14**	.13**	.20**	.20**	-	.21**
Ryff–Autonomy	.01	.10*	-.17**	-.12**	-.07	-.03	-.13**	-.05	-.07	-.05	-	-.02
Ryff–Environmental Mastery	.06	.07	-.23**	-.17**	-.05	-.05	-.11*	-.16**	-.12**	-.06	-	-.13**
Ryff–Personal Growth	.05	.12**	-.32**	-.24**	.03	-.07	-.16**	-.06	-.14**	-.07	-	-.10*
Ryff–Positive Relations with Others	.02	.08	-.32**	-.29**	-.08	-.15**	-.10*	-.07	-.12**	-.13**	-	-.18**
Ryff–Purpose in Life	.05	.05	-.17**	-.11*	-.05	-.05	-.16**	-.10*	-.06	-.07	-	-.09*
Ryff–Self-Acceptance	.09	.14**	-.30**	-.25**	-.11	-.14**	-.07	-.02	-.12**	-.11**	-	-.17**
Gratitude (GQ-6)	-.01	.11**	-.36**	-.25**	-.07	-.10*	-.15**	-.10*	.20**	-.11**	-	-.16**
Competitiveness & Status Seeking	1	1	.14**	.06	.14**	.11*	.29**	.24**	.16**	.09*	-	.28**
Degradation & Rejection	.14**	.06	1	1	.24**	.40**	.14**	.03	.47**	.67**	-	.63**
Emotional Inhibition & Deprivation	.14**	.11*	.24**	.40**	1	1	-.06	.01	.25**	.38**	-	.40**
Overprotection & Overindulgence	.29**	.24**	.14**	.03	-.06	.01	1	1	.03	-.05	-	.13**
Punitiveness	.16**	.09*	.47**	.67**	.25**	.38**	.03	-.05	1	1	-	.50**
Controlling	-	.28**	-	.63**	-	.40**	-	.13**	-	.50**	-	1

Note.

**. Correlation is significant at the 0.01 level (2-tailed), in bold

*. Correlation is significant at the 0.05 level (2-tailed).

s-EMBU–Swedish acronym for (‘My memories of upbringing’); CTQ-28 –Childhood Trauma Questionnaire; PARQ–Parental Acceptance-Rejection Questionnaire (PARQ); PARQ–Personality Assessment Questionnaire; DASS-21 –Depression Anxiety Stress Scale; Ryff–Ryff Scales of Psychological Well-Being; GQ-6 –Gratitude Questionnaire.

CSS–Competitiveness & Status Seeking; DR–Degradation & Rejection; EID–Emotional Inhibition & Deprivation; OO–Overprotection & Overindulgence; PU–Punitiveness; CTL–Controlling.

### Phase 3 confirmatory factor analysis and psychometric testing

The seven factors for the ratings of the fathers that were tested on the Jakarta sample did not secure the minimum CFA fit indices values. As such, items from the EFA with high item-to-item correlations that were statistically significant were also identified, 12 such items (labeled “R”) for the ratings of fathers and three for the mothers, as shown in S3 Table. These items caused correlated measurement errors and problems in obtaining an adequate fit in the CFA [65, 80] and were therefore removed. While removing these items improved the fit indices, the values of the CFA fit indices were still not within the minimum cut off values for a good fit. Therefore, the factor structure was further modified by the removal of one subscale at a time until adequate fit index values were secured. The CFA process was therefore used as a tool not just to confirm a factor structure but also to trim items from a scale, as recommended by Netemeyer et al. [80]. For ratings of fathers, three factors with generally the lowest loadings were targeted for removal–Intrusiveness and Exploitation, Undependability and Irresponsibility, and Dependency and Alienation. For ratings of mothers, three factors were targeted for removal–Undependability and Irresponsibility, Fear of Harm and Illness, and Controlling (see factor loadings in S3 Table). For ratings of fathers, adequate fit indices were obtained from a model with five subscales and 20 items. Likewise for the ratings of the mothers, an adequate fit was obtained from a model with six subscales and 33 items (see Table 2). Both Young’s theoretical 17-factor model [6] and Sheffield’s nine-factor model [19] were tested again on this Jakarta sample as a reference point for the other more robust models under consideration. Not surprisingly, a poor fit resulted, as it did in Phase 1. The items selected for the ratings of fathers and mothers to form the final shorter version known as YPI-R2 (Fathers) and YPI-R2 (Mothers) and were marked “✓”as indicated in S3 Table. Both these factor structures were then tested on another independent sample, USA, when it became available at a later time, and again, a reasonable fit was obtained (YPI-R2 (Fathers) USA, χ^2^ = 311.71, *df* = 160, χ^2^/*df =* 1.95, RMSEA = .068 [0.057, 0.079], CFI = .94, TLI = .93; and YPI-R2 (Mothers) USA, χ^2^ = 941.34, *df* = 480, χ^2^/*df =* 1.96, RMSEA = .067 [0.061, 0.073], CFI = .93, TLI = .92). MGCFA of these reduced models for fathers and mothers was then conducted on the Jakarta (Eastern) and USA (Western) samples, and partial invariance (Configural and Metric) [72] was demonstrated by both the ratings of fathers and mothers (see Table 3). Thus new factor structures, known as YPI-R2 (Fathers) and YPI-R2 (Mothers), were established, with five subscales common to both scales (Degradation and Rejection, Competitiveness and Status Seeking, Emotional Inhibition and Deprivation, Overprotection and Overindulgence, and Punitiveness). The additional subscale that had emerged only from the ratings of mothers was Controlling (see S3 Table). The average statistically significant correlation among the final subscales of YPI-R2 was .23 and .35 for ratings of fathers and mothers, respectively (see bottom of Table 1). The reliability values of these subscales for the ratings of fathers and mothers in all three samples (Manila, Jakarta and USA) exceeded the value of .60. The reliability, mean and SD values for YPI-R2 (Fathers) and YPI-R2 (Mothers) from all three samples are shown in S6 Table. These two scales were then subjected to psychometric scrutiny using the Manila sample that was used for EFA in Phase 2, and from which the factor structure was originally derived.

**Table 2:** Comparison of fit indices of various models using Jakarta sample (Fathers, n = 366; Mothers, n = 383).

Sample/model	χ^2^	*df*	*p*	χ^2^/*df*	CFI	TLI	RMSEA
**Fathers**							
Young's model	6290.46	2348	< .001	2.68	0.68	0.65	0.07
Sheffield	1924.02	593	< .001	3.24	0.77	0.75	0.08
7 factors Removal of Intrusiveness and Exploitation only	798.78	329	< .001	2.43	0.87	0.85	0.06
6 Factors—Removal of Intrusiveness and Exploitation and Undependability and Irresponsibility	617.05	237	< .001	2.60	0.89	0.87	0.07
6 Factors—Removal of Intrusiveness and Exploitation, and Dependency and Alienation	555.17	237	< .001	2.34	0.90	0.88	0.06
5 factors—Removal of Dependency and Alienation, Intrusiveness and Exploitation, and Undependability and Irresponsibility	387.83	160	< .001	2.42	0.92	0.90	0.06
**Mothers**							
Young's model	5191.38	2348	< .001	2.21	0.78	0.77	0.06
Sheffield	1881.43	593	< .001	3.17	0.80	0.78	0.08
7 factors—Removal of Undependability and Irresponsibility only	1587.93	644	< .001	2.47	0.89	0.88	0.06
6 factors—Removal Undependability and Irresponsibility, and Controlling	1321.95	512	< .001	2.58	0.88	0.87	0.06
6 factors—Removal Undependability and Irresponsibility, and Fear of Harm and Illness	1278.39	480	< .001	2.66	0.90	0.89	0.07
5 factors (only strong scales) without Undependability and Irresponsibility, Controlling, and Fear of Harm and Illness	1024.85	367	< .001	2.79	0.90	0.89	0.07

**Table 3:** Fit indices from multigroup CFA for YPI-R2 (Fathers) and YPI-R2 (Mothers) using Jakarta (n = 366, 383) and USA (n = 204, 214) samples.

Model	Number of parameters	*χ*^*2*^	*df*	*p*	*χ*^*2*^*/df*	CFI	TLI	RMSEA [90% CI]		
		(Δ*χ*^*2*^)*	(Δ*df*)*			(ΔCFI)	(ΔTLI)	(ΔRMSEA)	Comparison	Decision
**Fathers (5 factors 20 items)**										
Configural invariance	260	714.31	320	(< .001)	2.23	0.93	0.91	0.066 [0.059, 0.072]	-	Accepted
							
Metric invariance	245	794.41	335	(< .001)	2.37	0.91	0.90	0.069 [0.063, 0.076]	Configural vs. Metric	Accepted
		(104.37)	(15)	(< .001)		(0.012)	(0.009)	(0.003)		
Scalar invariance	170	1156.33	410	(< .001)	2.82	0.86	0.87	0.080 [0.075, 0.085]	Metric vs. Scalar	Rejected
		(456.56)	(75)	(< .001)		(0.054)	(0.032)	(0.011)		
**Mothers (6 factors 33 items)**										
Configural invariance	426	2206.88	960	(< .001)	2.30	0.92	0.91	0.066 [0.062, 0.070]	-	Accepted
							
Metric invariance	399	2414.06	987	(< .001)	2.45	0.90	0.90	0.070 [0.066, 0.073]	Configural vs. Metric	Accepted
		(281.79)	(27)	(< .001)		(0.013)	(0.010)	(0.001)		
Scalar invariance	273	3036.51	1113	(< .001)	2.73	0.87	0.88	0.076 [0.073, 0.079]	Metric vs. Scalar	Rejected
		(870.24)	(126)	(< .001)		(0.035)	(0.021)	(0.006)		
Acceptance criteria for indices						>0.9	>0.9	<0.06		
(differences)						(<0.01)	(<0.01)	(<0.015)		

Note.

*The chi-square difference test results of nested models using the scaled chi-square (Satorra & Bentler, 2010) are reported as results DIFFTEST command implemented in Mplus (Asparouhov & Muth´en, 2006).

### Construct validity

The average statistically significant correlation values of the YPI-R2 (ratings of fathers and mothers combined) with the s-EMBU, CTQ and PARQ were .30, .29, and .42, respectively. Specifically, the subscales of YPI-R2 (Fathers) and YPI-R2 (Mothers) correlated significantly with the closest theoretically linked construct of the other parenting subscales (see Table 1). For example, the Degradation and Rejection of the YPI-R2 correlated the highest in moderate strength with Rejection subscale of the s-EMBU. All subscales of the CTQ contained facets of Abuse and Neglect, while all the PARQ subscales contained facets of Acceptance-Rejection constructs. Not surprisingly, their highest correlation in moderate strength was also with YPI-R of Degradation and Rejection, and Punitiveness. Similarly, the YPI-R2 for Emotional Inhibition and Deprivation correlated the highest with the subscale for Warmth (negative direction) of the s-EMBU, Emotional Abuse (mothers), and Emotional Neglect of the CTQ, Warmth, and Indifference / Neglect (score reversed) of the PARQ. The Controlling subscale of the YPI-R2 also correlated mostly with the s-EMBU subscales of Rejection, and Overprotection. Other meaningful and moderate correlations were seen with subscales of YPI-R2 and these parenting instruments, thereby demonstrating construct validity.

### Convergent and divergent validity

The average statistically significant correlation values of the YPI-R2 with measures of PAQ, DASS-21, GQ-6, and Ryff’s scale (see Table 1) were .20, .19, .17, and .18, respectively. These correlations were low in strength but significant; the other established parenting scales of s-EMBU, CTQ and PARQ also showed similar strengths of correlations, as did the YPI-R2. Small effect sizes of .20, .19 and .22 were also evident in the psychometric testing of the established s-EMBU [36] with measures of neuroticism, extraversion and self-esteem, respectively. A study by Thimm [4] showed further significant correlations between s-EMBU with measures of personality disorder symptoms and depression, with values of *r* = .26 and .22, respectively. A work by Putnick et al. [81] also showed small but statistically significant correlation values of the PARQ with measures of child adjustment ranging from .06 to .14. Thus it is not unusual for measures of past parenting patterns to result in small effect sizes with other measures such as emotional distress, personality dispositions, and well-being. The subscale of Degradation and Rejection of the YPI-R2 showed the highest positive correlations with all three subscales of DASS-21, revealing the susceptibility of people with this negative parenting pattern of the YPI-R2 to emotional distress. YPI-R2 subscales also showed meaningful negative correlations with a measure of positive well-being (Ryff’s scale of Psychological Well-Being) and gratitude as shown in Table 1.

For further evidence of convergent validity, the YPI-R2 (Fathers) and YPI-R2 (Mothers) scales correlated statistically significantly with the 18 EMSs in the USA sample in the same direction (see Table 4). It was clear that many of the EMSs had meaningful statistically significant associations with more than one subscale in the YPI-R2. The EMS of Social Isolation had significant correlations with the subscale of Degradation and Rejection in the YPI-R2 as well as with the Controlling subscale of YPI-R2 (Mothers). This showed that negative parenting patterns are associated with the EMS of Social Isolation, contrary to the hypothesis of Young et al. [6] that this EMS was associated only with external family environment.

**Table 4:** Phase 3– Pearson's correlation matrix of the YPI-R2 (Fathers) and YPI-R2 (Mothers) with YSQ-S3 using USA sample (n = 204, 214).

	CSS		DR		EID		OO		PU		CTL	
F: Father / M: Mother	F	M	F	M	F	M	F	M	F	M	F	M
Abandonment / Instability	.03	.01	.19**	.26**	.12	.19**	.18*	.18**	.15*	.20**	-	.33**
Approval-Seeking / Recognition-Seeking	.15*	.03	.09	.10	.03	.12	.18*	.21**	.15*	.05	-	.17*
Defectiveness / Shame	-.02	-.04	.11	.28**	.15*	.20**	.02	.06	.01	.18**	-	.23**
Dependence / Incompetence	.00	-.02	.21**	.15*	.00	.04	.14*	.23**	.05	.17*	-	.09
Emotional Deprivation	-.01	.01	.10	.17*	.15*	.20**	.11	.12	.00	.08	-	.18**
Emotional Inhibition	.04	.01	.05	.10	.16*	.18**	.09	.07	.02	.04	-	.11
Enmeshment / Undeveloped Self	.12	.14*	.07	.09	.01	.04	.38**	.42**	.06	.12	-	.23**
Entitlement / Grandiosity	.02	.09	.01	.07	.14*	.14*	.15*	.18**	.08	.04	-	.16*
Failure	.08	-.12	.22**	.11	.04	.09	.03	.18**	.01	.07	-	.10
Insufficient Self-Control / Self-Discipline	.05	-.09	.08	.02	.01	.09	.21**	.26**	.02	.06	-	.03
Mistrust / Abuse	.06	.08	.11	.21**	.11	.07	.16*	.03	.19**	.24**	-	.29**
Negativity / Pessimism	.08	.13	.09	.24**	.10	.18*	.20**	.16*	.02	.18**	-	.31**
Punitiveness	.12	.14*	.20**	.21**	.09	.17*	.12	.10	.20**	.22**	-	.16*
Self-Sacrifice	.15*	.13	.26**	.17*	.12	.09	.12	.02	.20**	.25**	-	.22**
Social Isolation / Alienation	.02	-.05	.16*	.19**	.07	.11	.07	.03	.07	.13	-	.19**
Subjugation	.13	.03	.20**	.20**	.03	.14*	.17*	.16*	.10	.19**	-	.18**
Unrelenting Standards / Hypercriticalness	.19**	.27**	.18**	.22**	.19**	.19**	.17*	.05	.20**	.20**	-	.28**
Vulnerability to Harm or Illness	-.07	.06	.05	.15*	-.06	.10	.11	.14*	.05	.17*	-	.25**

Note.

**. Correlation is significant at the 0.01 level (2-tailed), in bold

*. Correlation is significant at the 0.05 level (2-tailed).

YSQ-S3 –Young Schema Questionnaire 3 Short form; CSS–Competitiveness & Status Seeking; DR–Degradation & Rejection; EID–Emotional Inhibition & Deprivation; OO–Overprotection & Overindulgence; PU–Punitiveness; CTL–Controlling.

As evidence for divergent validity, the z-test proposed by Steiger [76] showed that differences in correlations between most concordant subscales in the YPI-R2 and s-EMBU were statistically and significantly higher than differences in correlations with less concordant subscales of both measures (see S7 and S8 Tables). The average statistically significant correlation value for the ratings of the fathers with subscales of the YPI-R2 that were most concordant with subscales of the s-EMBU, and those less so, were .45 and .23, respectively. For the ratings of the mothers, these values were .47 and .26, respectively (see S9 Table).

### Incremental validity

The values of skewness and kurtosis and inspection of Q-Q plot showed that the distribution of data for some of the dependent variables deviated from normality, but given the large sample size > 200 (*n* = 520, 538) and the use of a conservative *p* value (< .001), the effects of non-normality were minimized [82]. Hierarchical multiple regression was conducted in the following steps: Step 1, Gender; Step 2, the subscales from three established parenting instruments (i.e., PARQ, s-EMBU and CTQ); and Step 3, the subscales of YPI-R2 (Fathers). The same steps were repeated for the YPI-R2 (Mothers) subscales. Significant evidence for incremental validity was demonstrated in tests in which the combined effects of both the YPI-R (Fathers) and YPI-R (Mothers) accounted for additional highly statistically significant variance greater than the minimum recommended by Hunsley and Meyer [77] of Δ*R*^*2*^ = .0225 (or 2.25%), over and above that contributed by gender and the three established parenting scales, in 12 out of 17 of the dependent variables (see Table 5).

**Table 5:** Phase 3 –Hierarchical regression analysis of YPI-R2 predicting GQ-6, DASS-21, PAQ and Ryff's well-being using Manila sample (n = 520, 538).

		Fathers			Mothers	
	R^2^	ΔR^2^	ΔF	R^2^	ΔR^2^	ΔF
**Gratitude (GQ-6)**						
*Step 1*: Gender	.02	.02**	7.97	.01	.01**	7.58
*Step 2*: All s-EMBU, CTQ and PARQ Parenting Subscales	.23	.21***	11.56	.19	.18***	9.70
*Step 3*: All YPI-R2 (Negative) Subscales	.28	.05***	6.58	.24	.04***	4.71
**DASS-21—Anxiety**						
*Step 1*: Gender	.01	.01	3.42	.01	.01	3.10
*Step 2*: All s-EMBU, CTQ and PARQ Parenting Subscales	.15	.14***	6.81	.11	.11***	5.34
*Step 3*: All YPI-R2 (Negative) Subscales	.19	.04***	5.12	.13	.02	2.01
**DASS-21—Depression**						
*Step 1*: Gender	.00	.00	.00	.00	.00	.02
*Step 2*: All s-EMBU, CTQ and PARQ Parenting Subscales	.18	.18***	8.96	.14	.14***	7.08
*Step 3*: All YPI-R2 (Negative) Subscales	.25	.08***	10.11	.19	.05***	5.46
**DASS-21—Stress**						
*Step 1*: Gender	.01	.01*	5.14	.01	.01*	4.27
*Step 2*: All s-EMBU, CTQ and PARQ Parenting Subscales	.18	.17***	8.97	.16	.15***	7.99
*Step 3*: All YPI-R2 (Negative) Subscales	.20	.02	2.08	.18	.02	1.73
**PAQ Hostility/Aggression**						
*Step 1*: Gender	.02	.02**	8.91	.02	.02**	7.94
*Step 2*: All s-EMBU, CTQ and PARQ Parenting Subscales	.27	.25***	14.17	.27	.25***	14.89
*Step 3*: All YPI-R2 (Negative) Subscales	.27	.01	.90	.28	.01	1.27
**PAQ Dependency**						
*Step 1*: Gender	.01	.01**	7.07	.02	.02**	8.00
*Step 2*: All s-EMBU, CTQ and PARQ Parenting Subscales	.06	.04*	1.96	.04	.03	1.25
*Step 3*: All YPI-R2 (Negative) Subscales	.07	.01	1.50	.05	.01	.72
**PAQ Negative Self-Esteem**						
*Step 1*: Gender	.00	.00	2.04	.00	.00	1.60
*Step 2*: All s-EMBU, CTQ and PARQ Parenting Subscales	.23	.25***	12.33	.17	.17***	8.98
*Step 3*: All YPI-R2 (Negative) Subscales	.29	.06***	7.94	.22	.04***	4.81
**PAQ Negative Self-Adequacy**						
*Step 1*: Gender	.00	.00	.00	.00	.00	.09
*Step 2*: All s-EMBU, CTQ and PARQ Parenting Subscales	.19	.19***	9.87	.15	.15***	7.42
*Step 3*: All YPI-R2 (Negative) Subscales	.27	.08***	11.24	.21	.06***	6.63
**PAQ Emotional Unresponsive**						
*Step 1*: Gender	.02	.02**	7.90	.01	.01**	6.70
*Step 2*: All s-EMBU, CTQ and PARQ Parenting Subscales	.13	.12***	5.60	.12	.11***	5.49
*Step 3*: All YPI-R2 (Negative) Subscales	.18	.05***	5.53	.15	.03*	2.78
**PAQ Emotional Instability**						
*Step 1*: Gender	.00	.00	.76	.00	.00	1.48
*Step 2*: All s-EMBU, CTQ and PARQ Parenting Subscales	.16	.16***	8.01	.15	.15***	7.43
*Step 3*: All YPI-R2 (Negative) Subscales	.18	.02*	2.30	.17	.02	1.91
**PAQ Negative World View**						
*Step 1*: Gender	.00	.00	.32	.00	.00	.60
*Step 2*: All s-EMBU, CTQ and PARQ Parenting Subscales	.28	.27***	15.99	.24	.24***	13.67
*Step 3*: All YPI-R2 (Negative) Subscales	.31	.04***	5.40	.27	.03**	3.53
**Ryff- Autonomy**						
*Step 1*: Gender	.00	.00	.00	.00	.00	.26
*Step 2*: All s-EMBU, CTQ and PARQ Parenting Subscales	.08	.08***	3.47	.05	.05**	2.47
*Step 3*: All YPI-R2 (Negative) Subscales	.11	.04**	4.13	.08	.02	2.01
**Ryff- Environmental Mastery**						
*Step 1*: Gender	.01	.01	2.77	.01	.01	2.90
*Step 2*: All s-EMBU, CTQ and PARQ Parenting Subscales	.15	.14***	6.94	.13	.12***	6.11
*Step 3*: All YPI-R2 (Negative) Subscales	.19	.04***	4.87	.18	.05***	5.54
**Ryff- Personal Growth**						
*Step 1*: Gender	.00	.00	.35	.00	.00	.18
*Step 2*: All s-EMBU, CTQ and PARQ Parenting Subscales	.19	.19***	9.62	.14	.14***	7.29
*Step 3*: All YPI-R2 (Negative) Subscales	.24	.06***	7.67	.19	.04***	4.50
**Ryff- Positive Relations with Others**						
*Step 1*: Gender	.01	.01*	3.90	.01	.01	3.61
*Step 2*: All s-EMBU, CTQ and PARQ Parenting Subscales	.18	.17***	8.69	.17	.16***	8.51
*Step 3*: All YPI-R2 (Negative) Subscales	.23	.05***	6.59	.20	.03**	3.71
**Ryff- Purpose in Life**						
*Step 1*: Gender	.00	.00	.31	.00	.00	.32
*Step 2*: All s-EMBU, CTQ and PARQ Parenting Subscales	.07	.07***	2.95	.06	.06***	2.82
*Step 3*: All YPI-R2 (Negative) Subscales	.11	.04***	4.87	.08	.02	1.62
**Ryff- Self-Acceptance**						
*Step 1*: Gender	.00	.00	1.31	.00	.00	.94
*Step 2*: All s-EMBU, CTQ and PARQ Parenting Subscales	.19	.18***	9.57	.17	.17***	8.85
*Step 3*: All YPI-R2 (Negative) Subscales	.23	.04***	5.54	.20	.03**	3.00

Note.

* *p* ≤ .05

** *p* < .01

*** *p* < .001

## Discussion

In ST practice, the YSQ is used to identify the EMSs linked to a patient’s presenting problems. The YPI is used along with the YSQ-S3 to help identify the likely origin of these EMSs. The YPI was developed based on the assumption that each EMS originated from a corresponding unmet core emotional need resulting from a pattern of dysfunctional parenting. While the identification of the origin of EMSs plays a central role in both the conceptualization and treatment phases of ST, unlike the YSQ, the YPI did not meet current standards for development and validation.

The aim of this research study was to first investigate the factor structure of two previous models, one by Young et al. [6] and the other by Sheffield et al. [19], on a sample from Singapore. Following poor fit for both models, a strong initial item pool was developed for the YPI with the aim to derive a shorter and validated version of the instrument, to be called YPI-R2 (Fathers) and YPI-R2 (Mothers) for the ratings of fathers and mothers, respectively.

This process was conducted through the course of three separate phases. Phase 1 identified robust and weak subscales in the YPI through EFA on a Singapore sample. Based on this EFA result, in Phase 2, a significantly expanded item pool of 204 items was developed for the YPI to strengthen the weak subscales and include other parenting constructs that have emerged in clinical sessions but were not represented in the original YPI. This longer version of YPI was then subjected to EFA on an independent sample from Manila, Philippines, where the most salient items were selected for each factor. In Phase 3, the updated and shorter item pool was then subject to CFA on an Eastern sample from Jakarta, Indonesia. This factor structure was modified during CFA in order to obtain adequate fit indices, resulting in five factors comprising 20 items for the ratings of fathers, and six factors comprising 33 items for the mothers (For the YPI-R2 final scale, please see S1 File). These final structures were then tested on a USA sample when it became available, and again, adequate fit was obtained. Results from MGCFA also showed partial invariance for support of the factor structure across these two separate and independent samples, an Eastern (Jakarta), and a Western (USA). The scales were then tested for construct, convergent and incremental validity as well as its relationship with EMSs in the USA sample.

Construct validity was shown through significant correlations between subscales of the YPI-R2 (Fathers) and YPI-R2 (Mothers) with similar subscales of the three established parenting instruments: the s-EMBU, CTQ and PARQ. Evidence for convergent validity is seen from statistically significant negative correlations between the subscales of YPI-R2 (Fathers) and YPI-R2 (Mothers) with the positive trait of gratitude (GQ-6), measures of well-being (Ryff’s Psychological Scale of Well Being), and positive correlations with measures of emotional distress, and negative personality dispositions (PAQ). Incremental validity for the YPI-R2 (Fathers) and YPI-R2 (Mothers) were also demonstrated, as delineated by Hunsley and Meyer [77], for most of the dependent subscales (*p* < .001).

ST has postulated a link between the development of EMSs and the nature of the relationship between a child and caregivers. This link is supported by the results of this study, as seen from the significant correlations between the subscales of the YPI-R2 (Fathers) and YPI-R2 (Mothers) and the 18 EMSs in the USA sample (see Table 4). This mirrored the associations found between EASs and positive parenting constructs [15, 16].

The EMS of Social Isolation had clear associations with the parenting patterns of Degradation and Rejection, and Controlling, contrary to the hypothesis by Young et al. [6] that the development of this EMS was primarily due to external environment outside the family. Since each EMS was linked with several parenting patterns, it can be deduced that there was not a one-to-one correspondence between a specific type of negative parenting pattern and a specific EMS, as hypothesized by Young [83, 6]. The final combined scales of YPI-R2 (Fathers) and YPI-R2 (Mothers), known as YPI-R2, consisted of six subscales and 36 items, compared to the original YPI with 72 items. Of the 72 items making up the original YPI, only 15 were robust enough to be retained in the final YPI-R2 scale. The remaining 21 items were new and/or revised. The reduced number of items in the YPI-R2, the good psychometric validation, and invariance of the factor structure across Eastern and Western samples indicated significant improvements to the original YPI.

Findings from other research for decades have shown that negative parenting patterns across cultures are linked to negative developmental outcomes [84, 85, 86]. However, some of these receive more emphasis due to differing cultural norms. For example, literature has highlighted that Eastern parents are more likely to be less expressive and connected, and to value the opinion of others in the society more than their counterparts in the West [87, 88]. This pattern is partly reflected by constructs found in this study such as Emotional Inhibition and Deprivation as well as Disconnection and Rejection. By contrast, Western parents are more likely to protect and support children’s self-expression [87, 88], which can lead to overprotection and difficulty introducing healthy limits, which in turn is reflected by the scale Overprotection and Overindulgence. Thus culturally influenced parenting patterns that are viewed as normative can, in different ways, negatively influence parenting both in the East and the West, as seen by the negative and positive correlations of these scales with measures of well-being and ill-being, respectively (Table 1). The invariant factor structure of the YPI-R2 in both a Western and Eastern sample also shows the cross-cultural relevance of the YPI-R2. Therefore, these results show that parenting patterns that are harmful to both cultures should become important targets for parenting intervention; whether they be ST based or rooted in some other model.

There were limitations in this study, the first being that it was based solely based on nonclinical samples. It will therefore be important to test this instrument on clinical samples. The second was that the sample was based on those who were drawn to the workshop on parenting, possibly limiting generalizability of the results to individuals with these traits.

Whilst most of the subscales exhibited high internal consistency, one or two had lower values in two Asian samples, and this may attenuate correlation size if replicated (hence, results may be an under-estimate). However, low internal consistencies would count against our hypotheses that the scale has good psychometric properties, as the added error would decrease, not increase values, in the tests of reliability and validity (and hence lead to Type II, not Type I, error). Our YPI-R2 scale consistently showed good psychometric properties. The non-normality of some of the data for the dependent variables in the regression analysis may also have been a limitation, though the sample size was large, and a very conservative *p* value (< .001) was achieved in most of the regression models.

The contribution of the YPI-R2 is a significant step towards uncovering more nuanced past negative parenting experiences, given that most established and validated past parenting measures have only three or four subscales. Since it is unlikely that complicated parenting patterns can be adequately assessed by only a few subscales, an instrument such as the YPI-R2 with six subscales would be able to provide fresh insights into the nature of negative parenting, and to be used hand in hand with the YSQ-S3. These insights will be useful for those involved in ST practice and research as well as be more broadly applicable to all clinicians and researchers interested in a better understanding of the nature and impact of maladaptive parenting.

## Supporting information

S1 Table Early maladaptive schemas and their hypothesized links to early parenting patterns in the YPI, and core emotional needs. (DOCX)

S2 Table Socio-demographic characteristics of the participants in the Singapore (Phase 1); Manila (Phase 2 & Phase 3); Jakarta & USA samples (Phase 3). (DOCX)

S3 Table EFA of the initial item pool of the YPI with 204 items using Manila sample (Father, n = 520; Mother, n = 538). (DOCX)

S4 Table Inter-factor correlation for Fathers Manila negative parenting. (DOCX)

S5 Table Inter-factor correlation for Mothers Manila negative parenting. (DOCX)

S6 Table Reliability Coefficients (α), Mean (M) and Standard Deviation (SD) of the YPI-R2 (Fathers; 5 factors 20 items) and YPI-R2 (Mothers; 6 factors 33 items). (DOCX)

S7 Table Divergent validity of the YPI-R2 (Fathers) with s-EMBU (Fathers) using the Manila sample (n = 520–5 factors 20 items). (DOCX)

S8 Table Divergent validity of the YPI-R2 (Mothers) with s-EMBU (Mothers) using the Manila sample (n = 538–6 factors 33 items). (DOCX)

S9 Table Average correlation between YPI-R2 subscales and counterparts from s-EMBU subscales. (DOCX)

S1 File Young Parenting Inventory–Revised (YPI-R2) Questionnaire. (PDF)

S1 Supporting Information Dataset on Jakarta, Manila, Singapore, and USA. (ZIP)

S2 Supporting Information Manila Output. (ZIP)

S3 Supporting Information Missing values. (ZIP)

S4 Supporting Information MultiGroup Fathers and Mothers. (ZIP)

S5 Supporting Information Divergent validity. (ZIP)

S6 Supporting Information Legend of Questionnaires. (ZIP)
