# Supplementary material for: Psychometric validation of the Young Parenting Inventory - Revised (YPI-R2): Replication and Extension of a commonly used parenting scale in Schema Therapy (ST) research and practice
Source: PLoS One. 2018 Nov 7;13(11):e0205605. doi: 10.1371/journal.pone.0205605 (PMC6221272; doi:10.1371/journal.pone.0205605)
Supplement: S1 Table — (DOCX) [file pone.0205605.s001.docx]

S1 Table

*Early Maladaptive Schemas and their Hypothesized Links to Early Parenting Patterns in the YPI, and Core Emotional Needs*

| Core Emotional Needs | Unmet Core Emotional Needs | Early Maladaptive Schemas (EMSs) | Sample items of corresponding negative parenting patterns from YPI (2003) |
| --- | --- | --- | --- |
| Connection & Acceptance | Disconnection & Rejection | Mistrust / Abuse | 31. Abused me physically, emotionally, or sexually. |
|  |  |  | 43. Used me to satisfy his/her needs. |
|  |  | Defectiveness / Shame | 11. Criticized me a lot. |
|  |  |  | 24. Made me feel unloved or rejected. |
|  |  | Emotional Deprivation | 20. Spent time with and paid attention to me. |
|  |  |  | 61. Was warm and physically affectionate. |
|  |  | Social Isolation / Alienation* |  |
|  |  |  |  |
|  |  | Emotional Inhibition | 8. Had to have everything under control. |
|  |  |  | 71. Was private; rarely discussed his/her feelings. |
|  |  | Failure | 23. Treated me as if I was stupid or untalented. |
|  |  |  | 63. Expected me to be a failure in life. |
| Healthy Autonomy | Impaired Autonomy | Vulnerability to Harm or Illness | 10. Worried excessively that I would get hurt. |
|  |  |  | 44. Was a fearful or phobic person. |
|  |  | Dependence / Incompetence | 22. Made me feel I couldn't rely on my decisions or judgment. |
|  |  |  | 32. Treated me as if I were younger than I really was. |
|  |  | Enmeshment / Undeveloped Self | 7. We were so close that we understood each other almost perfectly. |
|  |  |  | 55. I felt that we would hurt each other if either of us went away from the other. |
|  |  | Abandonment / Instability | 30. Was moody, unpredictable, or an alcoholic. |
|  |  |  | 59. Withdrew or left me alone for extended periods. |
|  |  | Subjugation | 4. Treated me as if my opinions or desires didn't count. |
|  |  |  | 33. Everything had to be on his/her terms. |
|  |  | Negativity / Pessimism | 28. Worried a lot about the family's financial problems. |
|  |  |  | 58. Had a pessimistic outlook; often expected the worst outcome. |
| Reasonable | Impaired | Entitlement / Grandiosity | 6. Spoiled me, or was overindulgent, in many respects. |
| Limits | Limits |  | 50. Was demanding; expected to get things his/her way. |
|  |  | Insufficient Self-Control / Self-Discipline | 13. Provided very little discipline or structure for me. |
|  |  |  | 18. Set few rules or responsibilities for me. |
|  |  | Approval-Seeking / Recognition-Seeking | 9. Was concerned with social status and appearance. |
|  |  |  | 29. Placed strong emphasis on success and competition. |
| Realistic | Exaggerated | Unrelenting Standards / Hypercriticalness | 35. Expected me to do my best at all times. |
| Expectations | Expectations |  | 68. Had strict, rigid rules of right and wrong. |
|  |  | Punitiveness | 19. Would punish me when I did something wrong. |
|  |  |  | 53. Blamed people when things went wrong. |
|  |  | Self-Sacrifice | 5. Sacrificed his/her own needs for the sake of the family. |
|  |  |  | 48. Was unhappy a lot and relied on me for support and understanding. |

*Note*: *Items for Social Isolation / Alienation sub-scale were never developed in YPI.
