# Supplementary material for: Psychometric validation of the Young Parenting Inventory - Revised (YPI-R2): Replication and Extension of a commonly used parenting scale in Schema Therapy (ST) research and practice
Source: PLoS One. 2018 Nov 7;13(11):e0205605. doi: 10.1371/journal.pone.0205605 (PMC6221272; doi:10.1371/journal.pone.0205605)
Supplement: S2 Table — (DOCX) [file pone.0205605.s002.docx]

S2 Table

*Socio-Demographic Characteristics of the Participants in the Singapore (Phase 1); Manila (Phase 2 & Phase 3); Jakarta & USA Samples (Phase 3)*

|  | Categories | Phase 1 | Phase 2 & 3 | Phase 3 | Phase 3 |
| --- | --- | --- | --- | --- | --- |
|  |  | Singapore sample for EFA; *n* (%) | Manila sample  for EFA; *n* (%) | Jakarta sample  for CFA; *n* (%) | USA sample  for CFA; *n* (%) |
| Gender | Men | 252 (40.32) | 222 (39.93) | 154 (39.09) | 85 (39.72) |
|  | Women | 371 (59.36) | 327 (58.81) | 225 (57.11) | 129 (60.28) |
|  | Did not specify | 2 (0.32) | 7 (1.26) | 15 (3.81) | 0 (0.00) |
| Age (years) | 19 | 17 (2.72) | N. A. | N. A. | N. A. |
|  | 20-29 | 87 (13.92) | 38 (6.83) | 103 (26.14) | 84 (39.25) |
|  | 30-39 | 271 (43.36) | 235 (42.27) | 142 (36.04) | 42 (19.63) |
|  | 40-49 | 216 (34.40) | 228 (41.01) | 111 (28.17) | 38 (17.76) |
|  | >= 50 | 34 (5.44) | 49 (8.81) | 22 (5.58) | 50 (23.36) |
|  | Did not specify | 1 (0.16) | 6 (1.08) | 16 (4.06) | 0 (0.00) |
| Parenting Status | Non parent | 260 (41.60) | 104 (18.70) | 143 (25.72) | N. A. |
|  | Parent | 328 (52.48) | 437 (78.60) | 216 (38.85) | N. A. |
|  | Did not specify | 37 (5.92) | 15 (2.70) | 35 (35.43) | N. A. |
| Race | Chinese | 526 (84.16) | 3 (0.54) | 164 (80.51) | N. A. |
|  | Malay | 1 (0.16) | N. A. | N. A. | N. A. |
|  | Indian | 12 (1.92) | N.A. | 2 (2.38) | N. A. |
|  | Indonesian | N. A. | N.A. | 197 (0.79) | N. A. |
|  | Filipino | N. A. | 540 (97.12) | 4 (14.42) | N. A. |
|  | Caucasian / White | N. A. | 1 (0.18) | 1 (0.32) | 91 (42.52) |
|  | Black | N. A. | N. A. | N. A. | 85 (39.72) |
|  | Latino | N. A. | N. A. | N. A. | 15 (7.01) |
|  | Asian | N. A. | N. A. | N. A. | 9 (4.21) |
|  | Others | 83 (13.28) | 3 (0.54) | 6 (1.43) | 13 (6.07) |
|  | Did not specify | 3 (0.48) | 9 (1.62) | 20 (0.16) | 1 (0.47) |
| Educational Qualification | Masters Degree & above | N. A. | N. A. | N. A. | 52 (24.30) |
|  | Postgraduate | N. A. | N. A. | N. A. | 11 (5.14) |
|  | Bachelors Degree | N. A. | N. A. | N. A. | 87 (40.65) |
|  | High School | N. A. | N. A. | N. A. | 45 (21.03) |
|  | Others | N. A. | N. A. | N. A. | 17 (7.94) |
|  | Did not specify | N. A. | N. A. | N. A. | 2 (0.93) |
| Nationality | Singaporean | 425 (68.00) | N. A. | N. A. | N. A. |
|  | Non-Singaporean | 198 (31.68) | N. A. | N. A. | N. A. |
|  | Malaysian | N. A. | N. A. | 1 (0.25) | N. A. |
|  | Indonesian | N. A. | N. A. | 366 (92.89) | N. A. |
|  | Indian | N. A. | N. A. | N.A. | N. A. |
|  | Filipino | N. A. | 546 (98.20) | 5 (1.27) | N. A. |
|  | Others | N. A. | 4 (0.72) | 5 (1.27) | N. A. |
|  | Did not specify | 2 (0.32) | 6 (1.08) | 17 (4.31) | N. A. |
| Total |  | 625 (100.0) | 556 (100.0) | 394 (100.0) | 214 (100.0) |
| Final Sample Size* | Fathers | 582 (93.12) | 520 (93.53) | 366 (92.89) | 204 (95.33) |
|  | Mothers | 617 (98.72) | 538 (96.76) | 383 (97.21) | 214 (100.0) |

*Note.* For each cell, data is presented as n (%); * Singapore: Father sample removed 43 who did not grow up with a father, Mother sample removed 8 who did not grow up with a mother; Manila: Father sample removed 36 who did not grow up with a father, Mother sample removed 18 who did not grow up with a mother; Jakarta: Father sample removed 28 who did not grow up with a father, Mother sample removed 11 who did not grow up with a mother; USA: Father sample removed 10 who did not grow up with a father, no further participants were removed for the mother sample.
