# Supplementary material for: Psychometric validation of the Young Parenting Inventory - Revised (YPI-R2): Replication and Extension of a commonly used parenting scale in Schema Therapy (ST) research and practice
Source: PLoS One. 2018 Nov 7;13(11):e0205605. doi: 10.1371/journal.pone.0205605 (PMC6221272; doi:10.1371/journal.pone.0205605)
Supplement: S3 Table — (DOCX) [file pone.0205605.s003.docx]

S3 Table

*EFA of the Initial Item Pool of the YPI with 204 Items Using Manila Sample (Father, n = 520; Mother, n = 538)*

|  |  | Father | | |  | Mother | | |
| --- | --- | --- | --- | --- | --- | --- | --- | --- |
| RQ1 Item No. | Item Description | Loading | Selected for YPI-R2 | Remarks |  | Loading | Selected for YPI-R2 | Remarks |
|  | ***Degradation & Rejection*** |  |  |  |  |  |  |  |
| 275 | Made me feel like the “black sheep” of the family. | .69 | ✓ |  |  | .65 | ✓ |  |
| 290 | Would call me names (like “stupid” or “idiot”) when I made mistakes. | .73 | R | High item correlation |  | .65 | ✓ |  |
| 313 | Saw me as lacking common sense. | .78 | ✓ |  |  | .72 | ✓ |  |
| 331 | Put me down and made me feel ashamed of myself if I didn’t do well. | .78 | R | High item correlation |  | .77 | ✓ |  |
| 384 | Saw me as having little to contribute. | .78 | ✓ |  |  | .68 | ✓ |  |
| 284 | Treated me as if I was stupid or untalented. | .81 | ✓ |  |  | .68 | ✓ |  |
| 346 | Saw me as not good at understanding the consequences of things. | .70 |  |  |  | .58 |  |  |
| 307 | Made me feel unloved or rejected. | .65 |  |  |  | .70 | ✓ |  |
| 386 | Criticized me a lot. | .68 |  |  |  | .75 | ✓ |  |
| 299 | Made me feel that almost nothing I did was quite good enough. | .41 |  |  |  | .46 |  |  |
| 336 | Was a fearful or phobic person. | .42 |  |  |  |  |  |  |
| 363 | Would ignore me or withdraw from me for long periods of time. | .43 |  |  |  | .48 |  |  |
| 388 | Made me feel guilty if I did not put his/her needs ahead of mine. | .43 |  |  |  |  |  |  |
| 355 | I never knew how s/he was going to treat me when s/he woke the next morning. | .46 |  |  |  | .40 |  |  |
| 314 | Often obsessed over minor decisions, because the consequences of making a mistake seemed so serious. | .47 |  |  |  | .45 |  |  |
| 394 | Was demanding; expected to get things his/her way. | .47 |  |  |  |  |  |  |
| 140 | Was never proud of me. | .48 |  |  |  | .45 |  |  |
| 227 | Treated me as if my opinions or desires didn't count. | .49 |  |  |  | .46 |  |  |
| 6 | Expected me to be a failure in life. | .49 |  |  |  | .47 |  |  |
| 253 | Would often compare my performance at school or sports unfavorably to others. | .52 |  |  |  | .51 |  |  |
| 407 | Would withdraw from me or reject me if I did not do what s/he thought I should. | .53 |  |  |  |  |  |  |
| 188 | Often told me there was something wrong with me. | .53 |  |  |  | .61 |  |  |
| 281 | Even if I did very well, s/he would focus on the mistakes or on things I didn’t do very well. | .53 |  |  |  | .65 |  |  |
| 243 | Down played my successes. | .54 |  |  |  | .48 |  |  |
| 393 | Even when things were good I was always waiting for the next outburst or bad reaction from him/her. | .55 |  |  |  | .65 |  |  |
| 399 | Did not seem to be interested in what I would do with my life. | .56 |  |  |  | .49 |  |  |
| 167 | Often told me I was bad. | .57 |  |  |  | .60 |  |  |
| 380 | Was unsure about my ability to reach challenging goals. | .57 |  |  |  | .49 |  |  |

S3 Table (Continued)

|  |  | Father | | |  | Mother | | |
| --- | --- | --- | --- | --- | --- | --- | --- | --- |
| RQ1 Item No. | Item Description | Loading | Selected for YPI-R2 | Remarks |  | Loading | Selected for YPI-R2 | Remarks |
|  | ***Degradation & Rejection (Continued)*** |  |  |  |  |  |  |  |
| 325 | I had to compete with my sibling(s) for his/her attention through outperforming them in sports or school. | .61 |  |  |  | .63 |  |  |
| 324 | Didn’t trust my ability to solve every day problems on my own that other children my age could. | .62 |  |  |  | .65 |  |  |
| 335 | Treated me as if I was not capable of coping well on my own. | .64 |  |  |  | .60 |  |  |
| 383 | Would make me look foolish or put me down in front of my friends or other adults. | .68 |  |  |  | .67 |  |  |
| 18 | Believed that I was better than other people. |  |  |  |  | -.45 |  |  |
| 120 | Treated me as if there was something wrong with me. |  |  |  |  | .49 |  |  |
| 202 | Made me feel to blame when things went wrong. |  |  |  |  | .47 |  |  |
| 293 | Was critical of my friends. |  |  |  |  | .43 |  |  |
| 303 | I felt like I needed to walk on eggshells around him/her. |  |  |  |  | .44 |  |  |
| 333 | I didn't expect him/her to respect me or take my feelings into account. |  |  |  |  | .46 |  |  |
|  | ***Competitiveness & Status Seeking*** |  |  |  |  |  |  |  |
| 98 | Placed strong emphasis on success and competition. | .78 | ✓ |  |  | .77 | ✓ |  |
| 64 | Believed that if I was smarter or more talented it made me superior to others who were less so. | .69 | ✓ |  |  | .64 | ✓ |  |
| 63 | Put a lot of emphasis on my getting good grades and getting ahead in life. | .68 | ✓ |  |  | .73 | ✓ |  |
| 110 | Believed that you are either a winner or a loser in life. | .63 | R | High item correlation |  | .47 | R | High item correlation |
| 52 | Was concerned with social status and appearance. | .57 | ✓ |  |  | .49 | R | High item correlation |
| 225 | Expected me to do my best at all times. | .53 | R | High item correlation |  | .52 | ✓ |  |
| 121 | Put more emphasis on competition and winning than getting along with others. | .54 | R | High item correlation |  | .53 |  |  |
| 330 | Drove me to excel at important tasks, couldn’t settle for “good enough”. | .54 |  |  |  | .49 |  |  |
| 236 | Believed I should to what ever it takes to come out ahead. | .54 |  |  |  | .47 |  |  |
| 160 | Was concerned with how my behavior would reflect on him/her in the eyes of others. | .51 |  |  |  | .41 |  |  |
| 152 | Saw it a “dog eat dog” world and believed that only the toughest and best survive. | .50 |  |  |  | .43 |  |  |
| 172 | Believed that those who come out ahead should be granted special privileges and not have to live by the same rules as others. | .46 |  |  |  |  |  |  |
| 109 | Believed that if someone had a lot of money and status that they would be happier than those who didn’t. | .46 |  |  |  |  |  |  |
| 387 | Put a lot of pressure on me to excel in important areas. | .45 |  |  |  |  |  |  |
| 198 | Was a perfectionist in many areas; things had to be “just so”. | .44 |  |  |  |  |  |  |
| 244 | Expected me to be the best in important areas; couldn’t accept my being second best. | .43 |  |  |  | .45 |  |  |
| 397 | Seemed to love me more or pay more attention to me when I excelled. | .42 |  |  |  |  |  |  |
| 7 | Had very high expectations for him/herself. |  |  |  |  | .47 |  |  |
| 39 | Was focused on my doing well at school and being responsible and had little interest in my having time for play and pursuing my own interests. |  |  |  |  | .43 |  |  |

S3 Table (Continued)

|  |  | Father | | |  | Mother | | |
| --- | --- | --- | --- | --- | --- | --- | --- | --- |
| RQ1 Item No. | Item Description | Loading | Selected for YPI-R2 | Remarks |  | Loading | Selected for YPI-R2 | Remarks |
|  | ***Emotional Inhibition & Deprivation*** |  |  |  |  |  |  |  |
| 273 | Was uncomfortable expressing affection. | .74 | ✓ |  |  | .65 | ✓ |  |
| 302 | Was private; rarely discussed his/her feelings. | .68 | ✓ |  |  | .55 | ✓ |  |
| 16 | Had a hard time being playful. | .59 | R | High item correlation |  | .54 | ✓ |  |
| 131 | Was uncomfortable expressing his/her feelings to others; even to people s/he knew well. | .58 | ✓ |  |  | .50 | ✓ |  |
| 319 | Did not seem comfortable playing with me. | .55 | R | High item correlation |  | .54 | R | High item correlations |
| 271 | Felt uncomfortable being silly and child-like. | .50 | ✓ |  |  | .44 | ✓ |  |
| 170 | Did not have a sense of humor. | .48 | R | High item correlation |  | .76 | ✓ |  |
| 154 | Was cold and distant. | .52 |  |  |  | .42 |  |  |
| 219 | Was not available for cuddling. | .52 |  |  |  | .49 |  |  |
| 254 | We were emotionally distant and had a hard time understanding each other. | .52 |  |  |  |  |  |  |
| 90 | I did not feel like I could go to him/her with questions about personal things; I would turn to friends or just keep it to myself. | .49 |  |  |  |  |  |  |
| 66 | Didn’t seem to be interested in spending time with me. | .44 |  |  |  |  |  |  |
| 112 | Was not interested in spending unplanned time together. | .41 |  |  |  |  |  |  |
| 85 | Was too self-conscious to show positive feelings to others even when s/he wanted to. | .41 |  |  |  |  |  |  |
| 233 | Took no interest in my friends. | .41 |  |  |  | .50 |  |  |
| 84 | Was a loner. |  |  |  |  | .51 |  |  |
| 169 | Had no interest in being part of a community. |  |  |  |  | .56 |  |  |
|  | ***Undependability & Irresponsibility*** |  |  |  |  |  |  | Scale removed - Low reliability |
| 187 | Was more focused on having fun and relaxing than keeping up with responsibilities. | .69 |  |  |  | .42 |  |  |
| 12 | Was an undisciplined person. | .63 |  |  |  | .57 |  |  |
| 392 | Spent too much money and was often in debt. | .58 |  |  |  | .44 |  |  |
| 104 | Was undependable and often did not follow through on plans we made. | .53 |  |  |  | .60 |  |  |
| 221 | Took money or a possession from me against my wishes to use for him/herself. | .44 |  |  |  | .52 |  |  |
| 53 | Was unable to handle many daily responsibilities, so I had to do more than my share. | .48 |  |  |  | .60 |  |  |
| 155 | Was an alcoholic or addicted to drugs. | .55 |  |  |  | .56 |  |  |
| 142 | Lied to me, deceived me, or betrayed me. | .45 |  |  |  | .51 |  |  |
| 91 | Abandoned me or left me on an emotional level when I was a child even though he/she was still physically present. | .45 |  |  |  |  |  |  |
| 56 | Provided very little discipline or structure for me. | .44 |  |  |  |  |  |  |
| 310 | Withdrew or left me alone for extended periods. | .43 |  |  |  |  |  |  |
| 19 | S/he dropped out of school and was not successful at work. | .42 |  |  |  |  |  |  |
| 10 | Never taught me the discipline necessary to succeed in school. |  |  |  |  | .50 |  |  |
| 45 | Left the house permanently when I was a child and did not keep in touch with me. |  |  |  |  | .62 |  |  |
| 58 | Would regularly break his/her promises to me. |  |  |  |  | .47 |  |  |
| 65 | I did not respect him/her. |  |  |  |  | .41 |  |  |
| 70 | Seemed to get pleasure out of hurting me. |  |  |  |  | .44 |  |  |
| 74 | Didn’t really want me to succeed. |  |  |  |  | .52 |  |  |
| 93 | Didn't seem to care what happened to me. |  |  |  |  | .43 |  |  |
| 156 | Used me or took advantage of me. |  |  |  |  | .62 |  |  |

S3 Table (Continued)

|  |  | Father | | |  | Mother | | |
| --- | --- | --- | --- | --- | --- | --- | --- | --- |
| RQ1 Item No. | Item Description | Loading | Selected for YPI-R2 | Remarks |  | Loading | Selected for YPI-R2 | Remarks |
|  | ***Overprotection & Overindulgence*** |  |  |  |  |  |  |  |
| 283 | Did a lot of things for me because s/he didn’t want me to get hurt. | .66 | R | High item correlation |  | .43 | ✓ |  |
| 9 | Did too many things for me instead of letting me do things on my own. | .66 | ✓ |  |  | .56 | ✓ |  |
| 123 | Overprotected me. | .62 | ✓ |  |  | .56 | ✓ |  |
| 95 | If I didn’t feel like doing a difficult or unpleasant task, I could usually get him/her to do it for me. | .61 | R | High item correlation |  | .50 | ✓ |  |
| 4 | Worried excessively that I would get hurt. | .45 | ✓ |  |  |  |  |  |
| 48 | Worried excessively that I would get sick. | .45 | ✓ |  |  |  |  |  |
| 371 | Spoiled me, or was overindulgent, in many respects. | .60 |  |  |  | .49 | ✓ |  |
| 305 | Treated me as if I was fragile. | .52 |  |  |  | .46 | ✓ |  |
| 277 | Made many decisions for me because s/he wanted to be sure things would turn out well. | .53 |  |  |  |  |  |  |
| 366 | Was over-involved in my life. | .53 |  |  |  | .42 |  |  |
| 49 | Would do my homework for me, if I felt overwhelmed by it. | .49 |  |  |  | .41 |  |  |
| 311 | I always let him/her make choices for me. | .46 |  |  |  |  |  |  |
| 50 | Has tried to live through me and did not allow me to be free to live my own life. | .44 |  |  |  |  |  |  |
| 288 | Treated me as if I were younger than I really was. | .43 |  |  |  |  |  |  |
|  | ***Punitiveness*** |  |  |  |  |  |  |  |
| 203 | Would punish me when I did something wrong. | .83 | ✓ |  |  | .65 | ✓ |  |
| 215 | Would punish me harshly when I did something wrong. | .77 | ✓ |  |  | .65 | ✓ |  |
| 148 | He/she relied more on punishment than praise and rewards. | .49 | R | High item correlation |  | .45 | ✓ |  |
| 3 | Abused me physically: did things like hitting me or throwing things at me. | .49 | ✓ |  |  | .60 | ✓ |  |
| 46 | Abused me verbally: did things like calling me names, screaming at me, swearing at me, or threatening me. | .45 | ✓ |  |  | .54 | ✓ |  |
| 129 | His/her punishments were often out of proportion with the “crime”. | .60 | R | High item correlation |  |  |  |  |
| 209 | When we disagreed s/he always needed to be right. | .59 |  |  |  |  |  |  |
| 184 | Everything had to be on his/her terms. | .56 |  |  |  |  |  |  |
| 168 | S/he believed that if you spared the rod you spoiled the child. | .53 |  |  |  |  |  |  |
| 265 | If I did what I wanted I was only asking from trouble with him/her. | .45 |  |  |  |  |  |  |
| 263 | Had to have everything under control. | .42 |  |  |  |  |  |  |
|  | ***Intrusiveness & Exploitation*** |  |  |  |  |  |  |  |
| 156 | Used me or took advantage of me. | .48 |  |  |  |  |  |  |
| 70 | Seemed to get pleasure out of hurting me. | .47 |  |  |  |  |  |  |
| 256 | Abused me sexually. | .44 |  |  |  |  |  |  |
| 74 | Didn’t really want me to succeed. | .41 |  |  |  |  |  |  |
|  | ***Dependency & Alienation*** |  |  |  |  |  |  |  |
| 8 | Was unhappy a lot and relied on me for support and understanding. | .54 |  |  |  |  |  |  |
| 214 | Discouraged me from inviting friends to our house. | .50 |  |  |  |  |  |  |
| 293 | Was critical of my friends. | .49 |  |  |  |  |  |  |
| 99 | Was often anxious and relied on me for reassurance and support. | .48 |  |  |  |  |  |  |
| 130 | Was (seemed to be) jealous of my friends. | .47 |  |  |  |  |  |  |
| 5 | Made me feel guilty if I did not share everything with him/her. | .41 |  |  |  |  |  |  |

S3 Table (Continued)

|  |  | Father | | |  | Mother | | |
| --- | --- | --- | --- | --- | --- | --- | --- | --- |
| RQ1 Item No. | Item Description | Loading | Selected for YPI-R2 | Remarks |  | Loading | Selected for YPI-R2 | Remarks |
|  | ***Controlling*** |  |  |  |  |  |  |  |
| 389 | Would make me feel guilty if I did not go along with him/her. |  |  |  |  | .69 | ✓ |  |
| 388 | Made me feel guilty if I did not put his/her needs ahead of mine. |  |  |  |  | .65 | ✓ |  |
| 394 | Was demanding; expected to get things his/her way. |  |  |  |  | .51 | ✓ |  |
| 349 | Put a lot of pressure on me to meet all of my responsibilities. |  |  |  |  | .40 | ✓ |  |
|  | ***Fear of Harm & Illness*** |  |  |  |  |  |  | Scales removed using CFA results |
| 178 | Worried about me or him/her being attacked. |  |  |  |  | .53 |  |  |
| 229 | Worried when making decisions that something terrible would happen if s/he made the wrong choice. |  |  |  |  | .47 |  |  |
| 257 | Worried about my developing a serious illness even though nothing serious was diagnosed by a physician. |  |  |  |  | .46 |  |  |
| 179 | S/he often thought that something bad was likely to happen to me. |  |  |  |  | .45 |  |  |
| 248 | Would worry that something terrible would happen if I made the wrong choice. |  |  |  |  | .43 |  |  |
|  | **Total Number of items** |  | **20** |  |  |  | **33** |  |
|  | **(Factors)** |  | **(5 factors)** |  |  |  | **(6 factors)** |  |
|  |  |  |  |  |  |  |  |  |

|  | Total Number of items selected (Fathers) |  | 20 |  |  |
| --- | --- | --- | --- | --- | --- |
|  | Total Number of items selected (Mothers) |  | 33 |  |  |
|  | Minus: Number of overlapped items |  | -17 |  |  |
|  | Final number of items |  | 36 |  |  |
