# Supplementary material for: Psychometric validation of the Young Parenting Inventory - Revised (YPI-R2): Replication and Extension of a commonly used parenting scale in Schema Therapy (ST) research and practice
Source: PLoS One. 2018 Nov 7;13(11):e0205605. doi: 10.1371/journal.pone.0205605 (PMC6221272; doi:10.1371/journal.pone.0205605)
Supplement: S4 Table — (DOCX) [file pone.0205605.s004.docx]

S4 Table

*Inter-factor correlation for Fathers Manila Negative Parenting*

| Factor | DR | CSS | EID | UDI | OO | PU | IE | DA |
| --- | --- | --- | --- | --- | --- | --- | --- | --- |
| Degradation & Rejection (DR) | 1 |  |  |  |  |  |  |  |
| Competitiveness & Status Seeking (CSS) | .32 | 1 |  |  |  |  |  |  |
| Emotional Inhibition & Deprivation (EID) | .58 | .16 | 1 |  |  |  |  |  |
| Undependability & Irresponsibility (UDI) | .53 | .05 | .55 | 1 |  |  |  |  |
| Overprotection & Overindulgence (OO) | .21 | .45 | -.07 | -.08 | 1 |  |  |  |
| Punitiveness (PU) | .58 | .51 | .47 | .32 | .30 | 1 |  |  |
| Intrusiveness & Exploitation (IE) | -.06 | -.26 | -.15 | .09 | -.13 | -.24 | 1 |  |
| Dependency & Alienation (DA) | .60 | .40 | .46 | .34 | .39 | .54 | -.18 | 1 |

*Note.* Extraction Method: Principal Axis Factoring; Rotation Method: Promax with Kaiser Normalization.
