# Supplementary material for: Psychometric validation of the Young Parenting Inventory - Revised (YPI-R2): Replication and Extension of a commonly used parenting scale in Schema Therapy (ST) research and practice
Source: PLoS One. 2018 Nov 7;13(11):e0205605. doi: 10.1371/journal.pone.0205605 (PMC6221272; doi:10.1371/journal.pone.0205605)
Supplement: S5 Table — (DOCX) [file pone.0205605.s005.docx]

S5 Table

*Inter-factor correlation for Mothers Manila Negative Parenting*

| Factor | DR | CSS | UDI | EID | OO | FHI | PU | CTL |
| --- | --- | --- | --- | --- | --- | --- | --- | --- |
| Degradation & Rejection (DR) | 1 |  |  |  |  |  |  |  |
| Competitiveness & Status Seeking (CSS) | .26 | 1 |  |  |  |  |  |  |
| Undependability & Irresponsibility (UDI) | .63 | .03 | 1 |  |  |  |  |  |
| Emotional Inhibition & Deprivation (EID) | .64 | .24 | .61 | 1 |  |  |  |  |
| Overprotection & Overindulgence (OO) | .25 | .31 | .08 | .14 | 1 |  |  |  |
| Fear of Harm & Illness (FHI) | .25 | .31 | .21 | .26 | .34 | 1 |  |  |
| Punitiveness (PU) | .40 | .37 | .32 | .45 | .17 | .23 | 1 |  |
| Controlling (CTL) | .55 | .45 | .39 | .52 | .38 | .37 | .56 | 1 |
