# Supplementary material for: Psychometric validation of the Young Parenting Inventory - Revised (YPI-R2): Replication and Extension of a commonly used parenting scale in Schema Therapy (ST) research and practice
Source: PLoS One. 2018 Nov 7;13(11):e0205605. doi: 10.1371/journal.pone.0205605 (PMC6221272; doi:10.1371/journal.pone.0205605)
Supplement: S6 Table — (DOCX) [file pone.0205605.s006.docx]

S6 Table

*Reliability Coefficients* (α), *Mean (M) and Standard Deviation (SD) of the YPI-R2 (Fathers; 5 factors 20 items) and YPI-R2 (Mothers; 6 factors 33 items)*

|  | Manila Sample | | | | | |  | Jakarta Sample | | | | | |  | USA Sample | | | | | |
| --- | --- | --- | --- | --- | --- | --- | --- | --- | --- | --- | --- | --- | --- | --- | --- | --- | --- | --- | --- | --- |
|  | Fathers | | | Mothers | | |  | Fathers | | | Mothers | | |  | Fathers | | | Mothers | | |
| Factors | α | *M* | *SD* | α | *M* | *SD* |  | α | *M* | *SD* | α | *M* | *SD* |  | α | *M* | *SD* | α | *M* | *SD* |
| Competitiveness & Status Seeking | 0.73 | 3.16 | 1.05 | 0.74 | 3.63 | 1.03 |  | 0.62 | 3.32 | 1.02 | 0.66 | 3.71 | 0.99 |  | 0.79 | 2.82 | 1.32 | 0.78 | 3.23 | 1.19 |
| Degradation & Rejection | 0.76 | 1.81 | 0.84 | 0.87 | 1.94 | 0.87 |  | 0.75 | 2.12 | 0.90 | 0.87 | 2.27 | 0.96 |  | 0.82 | 1.84 | 1.10 | 0.91 | 1.81 | 1.03 |
| Emotional Inhibition & Deprivation | 0.66 | 3.27 | 1.04 | 0.69 | 3.08 | 0.87 |  | 0.59 | 3.45 | 1.07 | 0.69 | 3.07 | 0.90 |  | 0.75 | 3.52 | 1.36 | 0.84 | 2.83 | 1.24 |
| Overprotection & Overindulgence | 0.62 | 2.99 | 0.98 | 0.69 | 2.94 | 0.88 |  | 0.70 | 2.88 | 1.07 | 0.62 | 2.93 | 0.85 |  | 0.71 | 1.82 | 0.91 | 0.81 | 2.34 | 1.07 |
| Punitiveness | 0.79 | 2.54 | 1.15 | 0.84 | 2.61 | 1.14 |  | 0.77 | 2.46 | 1.10 | 0.83 | 2.53 | 1.11 |  | 0.76 | 2.40 | 1.13 | 0.80 | 2.50 | 1.13 |
| Controlling | N. A. | N. A. | N. A. | 0.83 | 2.51 | 1.06 |  | N.A. | N. A. | N.A. | 0.79 | 2.90 | 1.13 |  | N.A. | N. A. | N.A. | 0.79 | 2.54 | 1.24 |
