# Supplementary material for: Psychometric validation of the Young Parenting Inventory - Revised (YPI-R2): Replication and Extension of a commonly used parenting scale in Schema Therapy (ST) research and practice
Source: PLoS One. 2018 Nov 7;13(11):e0205605. doi: 10.1371/journal.pone.0205605 (PMC6221272; doi:10.1371/journal.pone.0205605)
Supplement: S7 Table — (DOCX) [file pone.0205605.s007.docx]

S7 Table

*Divergent Validity of the YPI-R2 (Fathers) with s-EMBU (Fathers) Using the Manila Sample (n=520 –5 Factors 20 Items)*

| Scale j | Scale k | Scale h | Correlation between Scale j and scale k (r_jk) | Correlation between Scale j and scale h (r_jh) | Correlation between Scale k and scale h (r_kh) | z-test for testing if H0: r_jk - r_jh = 0 | 2-tailed p |
| --- | --- | --- | --- | --- | --- | --- | --- |
| Degradation & Rejection | sEMBU-Rejection | sEMBU-Emotional warmth | 0.53 | -0.36 | -0.32 | 14.01 | <.01 |
| Degradation & Rejection | sEMBU-Rejection | sEMBU-Overprotection | 0.53 | 0.33 | 0.56 | 5.62 | <.01 |
| Degradation & Rejection | sEMBU-Emotional warmth | sEMBU-Overprotection | -0.36 | 0.33 | 0.03 | -12.02 | <.01 |
| Emotional Inhibition & Deprivation | sEMBU-Rejection | sEMBU-Emotional warmth | 0.15 | -0.34 | -0.32 | 7.19 | <.01 |
| Emotional Inhibition & Deprivation | sEMBU-Rejection | sEMBU-Overprotection | 0.15 | 0.10 | 0.56 | 1.22 | 0.22 |
| Emotional Inhibition & Deprivation | sEMBU-Emotional warmth | sEMBU-Overprotection | -0.34 | 0.10 | 0.03 | -7.54 | <.01 |
| Overprotection & Overindulgence | sEMBU-Rejection | sEMBU-Emotional warmth | 0.13 | 0.20 | -0.32 | -1.00 | 0.32 |
| Overprotection & Overindulgence | sEMBU-Rejection | sEMBU-Overprotection | 0.13 | 0.36 | 0.56 | -5.72 | <.01 |
| Overprotection & Overindulgence | sEMBU-Emotional warmth | sEMBU-Overprotection | 0.20 | 0.36 | 0.03 | -2.71 | <.01 |
| Punitiveness | sEMBU-Rejection | sEMBU-Emotional warmth | 0.56 | -0.32 | -0.32 | 13.89 | <.01 |
| Punitiveness | sEMBU-Rejection | sEMBU-Overprotection | 0.56 | 0.37 | 0.56 | 5.48 | <.01 |
| Punitiveness | sEMBU-Emotional warmth | sEMBU-Overprotection | -0.32 | 0.37 | 0.03 | -11.96 | <.01 |
| Competitiveness & Status Seeking | sEMBU-Rejection | sEMBU-Emotional warmth | 0.09 | 0.18 | -0.32 | -1.35 | 0.18 |
| Competitiveness & Status Seeking | sEMBU-Rejection | sEMBU-Overprotection | 0.09 | 0.24 | 0.56 | -3.60 | <.01 |
| Competitiveness & Status Seeking | sEMBU-Emotional warmth | sEMBU-Overprotection | 0.18 | 0.24 | 0.03 | -0.86 | 0.39 |
