# Supplementary material for: Psychometric validation of the Young Parenting Inventory - Revised (YPI-R2): Replication and Extension of a commonly used parenting scale in Schema Therapy (ST) research and practice
Source: PLoS One. 2018 Nov 7;13(11):e0205605. doi: 10.1371/journal.pone.0205605 (PMC6221272; doi:10.1371/journal.pone.0205605)
Supplement: S8 Table — (DOCX) [file pone.0205605.s008.docx]

S8 Table

*Divergent Validity of the YPI-R2 (Mothers) with s-EMBU (Mothers) Using the Manila Sample (n=538 –6 Factors 33 Items)*

| Scale j | Scale k | Scale h | Correlation between Scale j and scale k (r_jk) | Correlation between Scale j and scale h (r_jh) | Correlation between Scale k and scale h (r_kh) | z-test for testing if H0: r_jk - r_jh = 0 | 2-tailed p |
| --- | --- | --- | --- | --- | --- | --- | --- |
| Degradation & Rejection | sEMBU-Rejection | sEMBU-Emotional warmth | 0.62 | -0.46 | -0.38 | 17.87 | <.01 |
| Degradation & Rejection | sEMBU-Rejection | sEMBU-Overprotection | 0.62 | 0.33 | 0.51 | 8.27 | <.01 |
| Degradation & Rejection | sEMBU-Emotional warmth | sEMBU-Overprotection | -0.46 | 0.33 | 0.02 | -14.39 | <.01 |
| Competitiveness & Status Seeking | sEMBU-Rejection | sEMBU-Emotional warmth | 0.09 | 0.19 | -0.38 | -1.35 | 0.18 |
| Competitiveness & Status Seeking | sEMBU-Rejection | sEMBU-Overprotection | 0.09 | 0.26 | 0.51 | -4.04 | <.01 |
| Competitiveness & Status Seeking | sEMBU-Emotional warmth | sEMBU-Overprotection | 0.19 | 0.26 | 0.02 | -1.25 | 0.21 |
| Emotional Inhibition & Deprivation | sEMBU-Rejection | sEMBU-Emotional warmth | 0.30 | -0.38 | -0.38 | 10.07 | <.01 |
| Emotional Inhibition & Deprivation | sEMBU-Rejection | sEMBU-Overprotection | 0.30 | 0.14 | 0.51 | 3.74 | <.01 |
| Emotional Inhibition & Deprivation | sEMBU-Emotional warmth | sEMBU-Overprotection | -0.38 | 0.14 | 0.02 | -9.21 | <.01 |
| Overprotection & Overindulgence | sEMBU-Rejection | sEMBU-Emotional warmth | 0.05 | 0.15 | -0.38 | -1.42 | 0.16 |
| Overprotection & Overindulgence | sEMBU-Rejection | sEMBU-Overprotection | 0.05 | 0.27 | 0.51 | -5.33 | <.01 |
| Overprotection & Overindulgence | sEMBU-Emotional warmth | sEMBU-Overprotection | 0.15 | 0.27 | 0.02 | -2.07 | 0.04 |
| Punitiveness | sEMBU-Rejection | sEMBU-Emotional warmth | 0.62 | -0.35 | -0.38 | 15.61 | <.01 |
| Punitiveness | sEMBU-Rejection | sEMBU-Overprotection | 0.62 | 0.31 | 0.51 | 8.57 | <.01 |
| Punitiveness | sEMBU-Emotional warmth | sEMBU-Overprotection | -0.35 | 0.31 | 0.02 | -11.69 | <.01 |
| Controlling | sEMBU-Rejection | sEMBU-Emotional warmth | 0.51 | -0.29 | -0.38 | 12.30 | <.01 |
| Controlling | sEMBU-Rejection | sEMBU-Overprotection | 0.51 | 0.45 | 0.51 | 1.53 | 0.13 |
| Controlling | sEMBU-Emotional warmth | sEMBU-Overprotection | -0.29 | 0.45 | 0.02 | -13.54 | <.01 |
