# Supplementary material for: Psychometric validation of the Young Parenting Inventory - Revised (YPI-R2): Replication and Extension of a commonly used parenting scale in Schema Therapy (ST) research and practice
Source: PLoS One. 2018 Nov 7;13(11):e0205605. doi: 10.1371/journal.pone.0205605 (PMC6221272; doi:10.1371/journal.pone.0205605)
Supplement: S9 Table — (DOCX) [file pone.0205605.s009.docx]

S9 Table

*Average Correlation Between YPI-R2 Subscales and Counterparts from s-EMBU Subscales*

| Negative Parenting Subscales | Correlation with counterpart | |  | Average correlation with non-counterparts (absolute values) | |
| --- | --- | --- | --- | --- | --- |
|  | Fathers | Mothers |  | Fathers | Mothers |
| Degradation & Rejection | 0.53 | 0.62 |  | 0.35 | 0.42 |
| Competitiveness & Status Seeking | No counterparts | No counterparts |  | 0.17 | 0.18 |
| Emotional Inhibition & Deprivation | 0.34 | 0.38 |  | 0.13 | 0.22 |
| Overprotection & Overindulgence | 0.36 | 0.27 |  | 0.17 | 0.10 |
| Punitiveness | 0.56 | 0.62 |  | 0.35 | 0.33 |
| Controlling | -- | 0.48 |  | -- | 0.29 |
| Average | 0.45 | 0.47 |  | 0.23 | 0.26 |
