# Supplementary material for: Psychometric validation of the Young Parenting Inventory - Revised (YPI-R2): Replication and Extension of a commonly used parenting scale in Schema Therapy (ST) research and practice
Source: PLoS One. 2018 Nov 7;13(11):e0205605. doi: 10.1371/journal.pone.0205605 (PMC6221272; doi:10.1371/journal.pone.0205605)
Supplement: S1 File — (PDF) [file pone.0205605.s010.pdf]

Young Parenting Inventory - Revised (YPI-R2)

This questionnaire is for personal use only. After the questionnaire is filled out, you may keep the questionnaire for your own reference. However, photocopying, scanning, distribution, and/or reproduction of this questionnaire are not allowed without prior written permission. If additional copies are needed, please email John Louis at johnphiliplouis@gmail.com.

INSTRUCTIONS:
Listed below are statements that you might use to describe your parents and how they treated you. Please read each statement and decide how well it describes your parents. Choose the highest rating from 1 to 6 that describes your father and your mother when you were a child and **write the number** in the spaces before each statement. If someone substituted as your mother or father, please rate the scale for that person. Give your first impression and move on to the next item. Do not dwell on any item. If you did not have a mother or a father, leave the appropriate column blank.

RATING SCALE:

1= Completely untrue	4= Moderately true
2= Mostly untrue	5= Mostly true
3= Slightly more true than untrue	6= Describes him/her perfectly

**Item No**	**Father**	**Mother**	**Description**
1.			Abused me physically: did things like hitting me or throwing things at me.
2.			Worried excessively that I would get hurt.
3.			Did too many things for me instead of letting me do things on my own.
4.			Criticized me a lot.
5.			Had a hard time being playful.
6.			Abused me verbally: did things like calling me names, screaming at me, swearing at me, or threatening me.
7.			Worried excessively that I would get sick.
8.			Made me feel guilty if I did not put his/her needs ahead of mine.
9.			Put a lot of emphasis on my getting good grades and getting ahead in life.
10.			Was demanding; expected to get things his/her way.
11.			Believed that if I was smarter or more talented it made me superior to others who were less so.

Copyright © 2018 Louis, J. P., Wood, A., & Lockwood, G. *Young Parenting Inventory – Revised (YPI-R2)*. Unauthorized reproduction without written consent of the author is prohibited. For more information, write to John Louis at email: johnphiliplouis@gmail.com

RATING SCALE:

1= Completely untrue	4= Moderately true
2= Mostly untrue	5= Mostly true
3= Slightly more true than untrue	6= Describes him/her perfectly

Item No	Father	Mother	Description
12.			Was uncomfortable expressing his/her feelings to others; even to people s/he knew well.
13.			Spoiled me, or was overindulgent, in many respects.
14.			Placed strong emphasis on success and competition.
15.			Would punish me when I did something wrong.
16.			Would make me feel guilty if I did not go along with him/her.
17.			If I didn't feel like doing a difficult or unpleasant task, I could usually get him/her to do it for me.
18.			Felt uncomfortable being silly and child-like.
19.			Made me feel like the "black sheep" of the family.
20.			Was uncomfortable expressing affection.
21.			Did a lot of things for me because s/he didn't want me to get hurt.
22.			Treated me as if I was stupid or untalented.
23.			Would punish me harshly when I did something wrong.
24.			Expected me to do my best at all times.
25.			Would call me names (like "stupid" or "idiot") when I made mistakes.
26.			Saw me as having little to contribute.
27.			Did not have a sense of humor.
28.			Put me down and made me feel ashamed of myself if I didn't do well.
29.			Saw me as lacking common sense.
30.			Was private; rarely discussed his/her feelings.
31.			Was concerned with social status and appearance.
32.			Overprotected me.
33.			He/she relied more on punishment than praise and rewards.
34.			Treated me as if I was fragile.
35.			Made me feel unloved or rejected.
36.			Put a lot of pressure on me to meet all of my responsibilities.

Copyright © 2018 Louis, J. P., Wood, A., & Lockwood, G. *Young Parenting Inventory – Revised (YPI-R2)*. Unauthorized reproduction without written consent of the author is prohibited. For more information, write to John Louis at email: johnphiliplouis@gmail.com

YPI-R2 SCORING KEY

Competitiveness & Status Seeking	9, 11, 14, 24, 31
Degradation & Rejection	4, 19, 22, 25, 26, 28, 29, 35
Emotional Inhibition & Deprivation	5, 12, 18, 20, 27, 30
Overprotection & Overindulgence	2, 3, 7, 13, 17, 21, 32, 34
Punitiveness	1, 6, 15, 23, 33
Controlling	8, 10, 16, 36

Listed below are the item numbers for each of the negative parenting schemas. Compute the total score and the mean score for each schema.

**Parenting Schemas**	**Parent**	**Rating**								**Total**	**Mean**
Competitiveness & Status Seeking	Father	9)	11)	14)	24)	31)					
	Mother	9)	11)	14)	24)	31)					
Degradation & Rejection	Father	4)	19)	22)	25)	26)	28)	29)	35)		
	Mother	4)	19)	22)	25)	26)	28)	29)	35)		
Emotional Inhibition & Deprivation	Father	5)	12)	18)	20)	27)	30)				
	Mother	5)	12)	18)	20)	27)	30)				
Overprotection & Overindulgence	Father	2)	3)	7)	13)	17)	21)	32)	34)		
	Mother	2)	3)	7)	13)	17)	21)	32)	34)		
Punitiveness	Father	1)	6)	15)	23)	33)					
	Mother	1)	6)	15)	23)	33)					
Controlling	Father	8)	10)	16)	36)						
	Mother	8)	10)	16)	36)						

TOTAL SCORE FOR FATHER: _______________ MOTHER: _______________

Copyright © 2018 Louis, J. P., Wood, A., & Lockwood, G. *Young Parenting Inventory – Revised (YPI-R2)*. Unauthorized reproduction without written consent of the author is prohibited. For more information, write to John Louis at email: johnphiliplouis@gmail.com
